# *Lb*DSF, the *Lysobacter brunescens* Quorum-Sensing System Diffusible Signaling Factor, Regulates Anti- *Xanthomonas* XSAC Biosynthesis, Colony Morphology, and Surface Motility

**DOI:** 10.3389/fmicb.2019.01230

**Published:** 2019-06-18

**Authors:** Jun Ling, Runjie Zhu, Pedro Laborda, Tianping Jiang, Yifan Jia, Yangyang Zhao, Fengquan Liu

**Affiliations:** ^1^Institute of Plant Protection, Jiangsu Academy of Agricultural Sciences, Jiangsu Key Laboratory for Food Quality and Safety-State Key Laboratory Cultivation Base of Ministry of Science and Technology, Nanjing, China; ^2^School of Life Sciences, Nantong University, Nantong, China; ^3^Institute of Life Sciences, Jiangsu University, Zhenjiang, China

**Keywords:** diffusible signaling factor, anti-*Xanthomonas* compound, colony morphology, surface motility, *Lysobacter brunescens*

## Abstract

*Lysobacter* species are emerging as novel sources of antibiotics, but the regulation of these antibiotics has not been thoroughly elucidated to date. In this work, we identified a small diffusible signaling factor (DSF) molecule (*Lb*DSF) that regulates the biosynthesis of a novel *Xanthomonas*-specific antibiotic compound (XSAC) in *Lysobacter brunescens* OH23. *Lb*DSF was isolated from the culture broth of *L. brunescens* OH23, and the chemical structure of the molecule was determined by NMR and MS. The *Lb*DSF compound induced GUS expression in a reporter strain of *Xanthomonas campestris* pv. *campestris* FE58, which contained the *gus* gene under the control of a DSF-inducible *engXCA* promoter. *Lb*DSF production was found to be linked to the enoyl-CoA hydratase RpfF and dependent on the two-component regulatory system RpfC (hybrid sensor histidine kinase)/RpfG (response regulator), and *Lb*DSF production was increased 6.72 times in the Δ*rpfC* compared to wild-type OH23. *Lb*DSF-regulated XSAC production was dramatically decreased in Δ*rpfF*, Δ*rpfC*, and Δ*rpfG*. Additionally, a significant reduction in surface motility and a number of changes in colony morphology was observed in the Δ*rpfF*, Δ*rpfC*, and Δ*rpfG* compared to the wild-type OH23. The exogenous *Lb*DSF significantly increased XSAC production in wild-type OH23 and recovered the XSAC biosynthetic ability in Δ*rpfF*. Taken together, these results showed that *Lb*DSF is a fatty-acid-derived DSF that positively regulates XSAC biosynthesis, cell morphology, and surface motility. Moreover, the RpfC/RpfG quorum-sensing signal transduction pathway mediates XSAC biosynthesis. These findings may facilitate antibiotic production through genetic engineering in *Lysobacter* spp.

## Introduction

The gliding Gram-negative *Lysobacter* bacteria are ubiquitous freshwater and soil microorganisms; because they are fast-growing, simple to maintain, and genetically amenable for bioengineering, they are a promising source of novel bioactive natural antibiotics (Christensen and Cook, 1978; Xie et al., 2012). Some *Lysobacter* species have been demonstrated to be capable of producing antibiotics, including cyclodepsipeptides, cyclic lipodepsipeptides, cephem-type β-lactams, polycyclic tetramate macrolactams, phenazines, and lactivicins, which can inhibit the growth of plant pathogens.

Lysobactin is a cyclodepsipeptide that was first reported by O’Sullivan et al. (1988). The structure of lysobactin contains five natural amino acid residues and six non-proteinogenic amino acids, forming a macrocycle. Conversely, two families of cyclic lipodepsipeptides have been isolated from *Lysobacter* species (Kato et al., 1997; Hashizume et al., 2001; Zhang et al., 2011), both of which contain a β-hydroxyl fatty acid linked to the peptide moiety. The WAP-8294A family consists of 12 amino acid macrocycles, whereas the tripopeptin family is formed by eight amino acid cycles and a branched acyl group varying in length from 11 to 16 carbons (Xie et al., 2012). Cephem-type β-lactam antibiotics include cephalosporins, cephabacins, and cephamycins, but only cephabacins have been isolated from *Lysobacter* species (Harada et al., 1984; Ono et al., 1984). Cephabacins contain a cephem nucleus, which is linked to a non-ribosomal peptide by an ester linkage. Three different polycyclic tetramate macrolactams, xanthobacin, dihydromaltophilin (HSAF), and maltophilin, have been isolated from *Lysobacter gummosus* OH17 and *Lysobacter enzymogenes* OH11. All three of these polycyclic tetramate macrolactams contain γ-butyrolactam, which is involved in the macrocyclic structure (Meyers et al., 1985; Lou et al., 2011; Qian et al., 2013; Wang et al., 2013). *Lysobacter* cephabacins were shown to employ a novel mode of antibacterial action. These cephabacins specifically interfere with the biosynthesis of sphingolipids by targeting ceramide synthase, which causes thickening of the cell wall due to the accumulation of sphingolipid promoters that increase the degradation of chitin and block the elongation of the hyphal tips (Li et al., 2009). Several phenazines, including myxin and iodinin, have been found in *Lysobacter antibioticus* OH13 (Zhao et al., 2016). These phenazines differ not only in the presence of an *N*-oxide bond but also in the substituent at position 1 of the heterocycle. Finally, it is worth mentioning that lactivicin has a unique structure composed of a cycloserine ring linked to a γ-lactone ring (Harada et al., 1986). HSAF was shown to have a wide range of biological activities, including antibiotic, antifungal, and anticancer activities. Our research group also described a new cyclic lipodepsipeptide, WAP-8294A2, from *L. enzymogenes*, which exhibits strong inhibitory activity against methicillin-resistant *Staphylococcus aureus* (Zhang et al., 2011). WAP-8294A2 is currently in phase I/II clinical studies. WAP-8294A2 is produced by a large non-ribosomal peptide synthetase complex of 12 modules, which forms the linear assembly of amino acids. The biosynthetic pathway leading to the formation of 6 bioactive phenazines in *L. antibioticus*, starting from chorismic acid, was also reported by our research group (Zhao et al., 2016). After isolation of the compounds using reverse-phase HPLC, it was demonstrated that these phenazines exhibited strong activity against several bacteria, including *Escherichia coli*, *Bacillus subtilis*, and *Xanthomonas* spp.

In some prokaryotic systems, bacteria produce exogenous chemical signaling molecules to monitor population density and regulate a wide range of biological functions, such as secondary metabolite biosynthesis, biofilm formation, colony morphology, surface motility, and virulence (Abisado et al., 2018). LuxI/LuxR-type quorum-sensing systems are very common in Gram-negative bacteria, where they regulate the expression of genes through small chemical auto-inducers (Van Houdt et al., 2007). LuxI proteins are responsible for the synthesis of *N*-acyl homoserine lactone (AHL) quorum-sensing signals, and LuxR proteins are considered to be the main regulatory component of AHL quorum-sensing systems. These two proteins share two conserved regions, an AHL-binding domain and a DNA-binding domain (Maddocks and Oyston, 2008). Another group of quorum-sensing signals, the diffusible signaling factor (DSF) family, has recently been reported in a range of plant and human bacterial pathogens, including *Xanthomonas campestris* pv. *campestris* (*Xcc*), *Xylella fastidiosa*, *Stenotrophomonas maltophilia*, and *L. enzymogenes* (Barber et al., 1997; Deng et al., 2011; Robert and Dow, 2011; Han et al., 2015). In the DSF system, the putative enoyl-CoA hydratase RpfF is responsible for the synthesis of the DSF, and the RpfC/RpfG two-component system is involved in sensing and transducing DSF signals through a conserved phosphorelay mechanism (Holly et al., 2000). Different types of DSF signal molecules have been identified by electrospray ionization mass spectrometry (ESI-MS), gas chromatography, and nuclear magnetic resonance (NMR) analysis, such as *cis*-11-methyl-2-dodecenoic acid (DSF), *cis*-dodecenoic acid (BDSF), *cis*-11-methyldodeca-2,5-dienoic acid (CDSF), and 13-methyltetradecanoic acid (Wang et al., 2004; He et al., 2010; Han et al., 2015). Functional analysis of *rpfF* and *rpfC* mutants in different bacterial species suggests that the general role of the DSF-signaling system in virulence modulation is conserved, but the regulatory mechanisms and DSF-dependent traits may differ among taxa (Holly et al., 2000).

In this study, we isolated a novel *Lysobacter brunescens* strain, OH23, and active compounds from the culture supernatant of OH23 were shown to have strong specific activity against *Xanthomonas* species, whereas other bacteria and fungi, including *Pseudomonas syringae* pv. *glycinea*, *P. syringae* pv. *lachrymans*, *Acidovorax citrulli*, *E. coli*, *Erwinia amylovora*, *Botryosphaeria dothidea*, *Phytophthora capsica*, *Valsa ambiens* var. *Pyri*, and *Colletotrichum gloeosporioide*, remained unaltered (Supplementary Figure S1). We found that the genome of *L. brunescens* OH23 contained gene homologs to the regulation of pathogenicity factor (*rpf*) gene clusters of quorum-sensing genes from *L. enzymogenes* OH11. DSF signaling has been shown to be involved in the synthesis of metabolites with high pharmaceutical interest in *L. enzymogenes* OH11. This finding prompted us to investigate whether a DSF-dependent quorum-sensing signaling pathway was also responsible for the production of *Xanthomonas*-specific antibiotic compounds (XSAC). To this end, we developed single in-frame mutants of the homolog DSF genes *rpfF*, *rpfC*, and *rpfG* and tested their ability to both produce DSF signals and synthesize XSAC. Our results revealed that *L. brunescens* uses an autoregulatory mechanism similar to *L. enzymogenes* to control DSF biosynthesis, suggesting the production of a DSF-like molecule in *L. brunescens* (Holly et al., 2000). We characterized the *L. brunescens* DSF signal as 13-methyltetradecanoic acid and demonstrated that this molecule regulates XSAC biosynthesis, surface motility, and cell morphology through the RpfC/RpfG signaling pathway.

**Table 1:** Bacterial strains and plasmids used in this study.

Strain/plasmid	Description	Source or references
***Lysobacter brunescens***		
OH23	Wild-type symbiotic strain	Lab strain
OH23 Rif	Spontaneous Rif^R^ mutant of OH23, Rif^R^	
Δ*rpfF*	*rpfF* gene in-frame deletion mutant	This study
Δ*rpfG*	*rpfG* gene in-frame deletion mutant	This study
Δ*rpfC*	*rpfC* gene in-frame deletion mutant	This study
***Xanthomonas***		
*Xanthomonas campestris* pv. *campestris* FE58	DSF biosensor strain, Rif^R^, Tc^R^	Wang et al., 2004
*Xanthomonas oryzae* pv. *oryzae* PXO99^A^	Plant pathogen, causes bacterial leaf blight disease in rice	Steven et al., 2008
***E. coli***		
DH5α λpir	*supE44 Dlacu169 (f80 lacZDM15) hsdR17 recA1 endA1 gyrA96 thi-1 relA1* λpir	Lab strain
S17-1 λpir	Tp^R^ Sm^R^ *recA thi pro hsdR^−^*M^++^ *recA*::RP4-2-Tc::Mu Km::Tn7 λpir	Lab strain
**Plasmid**		
pJQ200SK	Suicide cloning vector, Gm^R^	Quandt and Hynes, 1993
pJQ-*rpfF*	pJQ200SK derivative carrying two flanking fragments of *rpfF*, Gm^R^	This study
pJQ-*rpfG*	pJQ200SK derivative carrying two flanking fragments of *rpfG*, Gm^R^	This study
pJQ-*rpfC*	pJQ200SK derivative carrying two flanking fragments of *rpfC*, Gm^R^	This study

## Materials and Methods

### Bacterial Strains, Vectors, and Culture Conditions

The bacterial strains and plasmids used in this study are listed in Table 1. *E. coli* DH5α λpir, K-12, and S17-1 λpir were grown in LB (10 g tryptone, 5 g yeast extract, and 10 g sodium chloride, pH 7.0–7.2, in 1 L of distilled water) at 37°C (Sambrook, 2001). *L. brunescens* OH23 (stored at China General Microbiological Culture Collection Center, Beijing, CGMCC No. 13677) and the genetically engineered Δ*rpfF*, Δ*rpfC*, and Δ*rpfG* mutants were grown in nutrient broth (NB) medium (5 g peptone, 1 g yeast extract, 3 g beef extract, and 10 g sucrose, pH 7.0–7.2, in 1 L of distilled water) or in nutrient broth–yeast extract–glucose (NYG) medium (5 g peptone, 3 g yeast extract, and 20 g glycerol, pH 7.0–7.2, in 1 L of distilled water) at 28°C (Atlas, 1997). *X. campestris* pv. *campestris* FE58, *Xanthomonas oryzae* pv. *oryzae* PXO99^A^, *X. oryzae* pv. *oryzae* RS105, *X. oryzae* pv. *oryzae* KACC10331, *X. campestris* pv. *campestris* 8004, *Xanthomonas axonopodis* pv. *glycines* 12-2, *P. syringae* pv. *glycinea* PG4180, *A. citrulli* DSM17060, *P. syringae* pv. *lachrymans* 814/98, and *E. amylovora* ATCC15580 were also grown in nutrient broth (NB) medium at 28°C. All solid media contained 1.5% agar, and antibiotics were added at the following concentrations: 20 μg/ml rifampicin and 8 μg/ml gentamicin for *L. brunescens*, 50 μg/ml rifampicin, and 10 μg/ml tetracycline for *X. campestris* pv. *campestris* FE58, and 20 μg/ml gentamicin for *E. coli*. Sucrose was added at a final concentration of 4% for the counter-selection of in-frame deletion strains.

**Table 2:** Primers used in this study.

Primer name	Sequence (5′ → 3′)	Amplicon size (bp)	Usage
*rpfF*-1	CGAATTCCTGCAGCCCGGGGGATCCGCTGCTGCCATCGCGCAAGC	535	*rpfF* deletion
*rpfF*-2	GGCATCAGGCCGACATGCGAGTGGAAGTAGAG		
*rpfF*-3	TCGCATGTCGGCCTGATGCCGATGATCACGGATCCGGACC	535	
*rpfF*-4	CTGCCGCGGCAGCGGCCGCTCTAGATCGCATCGATCGCTGC		
*rpfG*-1	CGAATTCCTGCAGCCCGGGGGATCCACCGGCCAGCAGACCCCAGGA	535	*rpfG* deletion
*rpfG*-2	GTGGGGCAATGGGCTGGAGTGAACGCGCTGCGCGCCGCCTAC		
*rpfG*-3	ACTCCAGCCCATTGCCCCACATGCGGATAC	535	
*rpfG*-4	CTGCCGCGGCAGCGGCCGCTCTAGACCTCGGTGTCGGCATCGACCG		
*rpfC*-1	TCCTGCAGCCCGGGGGATCCTCGAAGGCGACGCGTTGGGTG	528	*rpfC* deletion
*rpfC*-2	TGAACGCGTGATCATCGGCATCAGGCCGTGGCGGCATCG		
*rpfC*-3	GATGATCACGCGTTCACTCCAGCCCCGGCCGTTG	528	
*rpfC*-4	GCGGCAGCGGCCGCTCTAGAATGCCCGAGGACGAGGTCCG		
M13-F	TGTAAAACGACGGCCAGT		Confirmation of vector construction
M13-R	CAGGAAACAGCTATGACC		
RT-*pilA*_1_-F	AAGCCGAACGTCCAGATATC	127	*pilA_1_* expression detection
RT-*pilA*_1_-R	GGCTGGAATTCGAGGAATAC		
RT-*recA*-F	GTCACCGAAATCCTCTATGG	164	
RT-recA-R	GGGTTGTCCTTCATGTACTG		

### Generation of In-Frame Deletion Mutants

In-frame deletion plasmids were constructed by amplifying the flanking regions of specific genes by polymerase chain reaction (PCR) using Tks Gflex DNA Polymerase (TaKaRa Bio Inc., Kusatsu, Japan) and OH23 genomic DNA as the template according to the manufacturer’s instructions. Briefly, PCR amplification was performed using 30 PCR cycles consisting of denaturation at 98°C for 10 s, annealing at 55°C for 15 s, and elongation at 68°C for 1 min on a Bio-Red S1000 thermal cycler. The suicide vector pJQ200SK was digested with *Xba*I and *Bam*HI (Thermo Fisher Scientific), and the target PCR fragments were ligated into the suicide vector using In-Fusion HD Cloning Plus (TaKaRa Bio Inc.). The recombinant vectors were transformed into *E. coli* DH5αλpir and confirmed using the universal primers M13F/M13R. The resulting plasmids were introduced into *L. brunescens* by conjugation. The deletion mutants Δ*rpfF*, Δ*rpfC*, and Δ*rpfG* were selected for double homologous recombination events because the suicide vector contained a *sacB* counter-selectable marker (Quandt and Hynes, 1993). Finally, all mutants were confirmed by PCR using specific primers (Table 2).

### Growth Measurements

*Lysobacter brunescens* OH23 and the *rpf* mutants were cultured in NB medium at 28°C with shaking at 180 rpm until the OD_600_ was approximately 1.0 [which corresponds to approximately 10^9^ CFU/ml (Colony Forming Units/ml)]. Then, 1 ml of culture for each strain was transferred into 50 ml of new liquid NB medium. The cultures were then incubated at 28°C with shaking at 180 rpm. To measure the growth, the OD_600_ value was determined every 12 h for each culture using a BioPhotometer Plus (Eppendorf, Germany) until each culture reached stationary stage. Three replicates were performed for each treatment, and the experiment was repeated three times.

### Bioassay for DSF Activities in a DSF Reporter Strain

The fermentation and isolation of *Lb*DSF was assessed as previously described (Han et al., 2015). After extracting the DSF-dependent quorum-sensing signals in *L. brunescens* OH23, we performed a DSF bioassay, as described previously with modifications (Wang et al., 2004). Briefly, *L. brunescens* OH23 and the Δ*rpfF*, Δ*rpfC*, and Δ*rpfG* mutants were grown in NB liquid medium (25 L liquid medium in a CRJ-50D fermenter, twice, for a total of 50 L) at 28°C at 180 rpm until the culture reached 2 × 10^9^ CFU/ml (OD_600_ = approximately 2.0). The culture broth was centrifuged at 12,000 rpm for 10 min, and the supernatant was extracted with the same volume of ethyl acetate. The extracted organic phase was evaporated at 47°C, and the dry crude extract was partitioned using methanol and petroleum ether (100 ml each, three times). The petroleum ether phase was evaporated at 47°C, and the resulting oil (2.1 g) was dissolved in 10 ml of dimethyl sulfoxide (DMSO). The DSF-reporter strain, *Xcc* FE58, was grown in liquid NYG medium (5.0 g peptone, 3.0 g yeast extract, 20.0 g glycerol, and 1.0 L water, pH 7.2) for 2 days until the culture reached 3 × 10^9^ CFU/ml (OD_600_ = approximately 3.0).

The bioassay plates were prepared using the following steps. First, 0.8 g agarose powder was added to 100 ml 0.5 × NYG liquid medium. The medium was heated until the agarose was resolved, at which point 60 μl of X-gluc (60 mg/ml) and 2 ml of the reporter strain *Xcc* FE58 culture (3 × 10^9^ CFU/ml) were added into the NYG agarose medium (the medium was cooled to 42°C before use). This medium was used to prepare the 90-mm bioassay plates. Then, 5 μl of the DSF crude extract (10 mg crude extract resuspended in 200 μl of DMSO) from *L. brunescens* OH23, Δ*rpfF*, Δ*rpfC*, or Δ*rpfG* mutants was added to each well. The bioassay plates were incubated at 28°C for 24 h. The quantification of *Lb*DSF was assessed as previously described (Wang et al., 2004). In brief, DSF activity was confirmed by the presence of a blue halo around the well and measured using the formula DSF (unit/ml) = 0.134 × e ^(1.9919^*^W^*^)^, where *W* is the diameter of the blue halo zone and the width of the blue halo zone was increased while adding more exogenous *Lb*DSF (Figure 2B).

One unit of DSF is equivalent to a 1-cm-diameter blue halo zone formed by the addition of 0.18 ± 0.07 μg exogenous *Lb*DSF. We also added DMSO to the bioassay plate as a negative control. Three replicates were performed for each treatment, and the experiment was repeated three times.

### Isolation and Identification of *Lb*DSF

The crude DSF extract was purified using HPLC. The HPLC detection conditions were as follows: reverse-phase HPLC (Shimadzu MS-2020, Tokyo, Japan) at 210 nm using a C-18 column (250 × 4.6 mm, Phenomenex). The mobile phase was 80% methanol in H_2_O from 0 to 13 min, 100% methanol in H_2_O from 13 to 20 min, and 80% methanol in H_2_O from 20 to 30 min (H_2_O containing 0.04% trifluoroacetic acid). The fraction corresponding to *Lb*DSF appeared at 11.5 min.

Fractions containing the desired compound were evaporated to dryness under reduced pressure. The purified compound (4.3 mg) was dissolved in chloroform and characterized by mass spectrometry and NMR. High-resolution ESI-MS of the purified compound was performed in an AB (QTRAP 6500) instrument. The samples were directly infused into a mass spectrometer and analyzed in negative ion mode using a Turbo Ion Spray source. The NMR spectra (^1^H NMR and ^13^C NMR) were recorded in CD_3_OD using an AVANCE 600 (Bruker Company, Germany) instrument with a standard pulse program.

**^1^H NMR (600 MHz, CDCl_3_):** δ 2.35 (t, 2H, *J* = 7.8 Hz), 1.63 (qt, 2H, *J* = 7.2 Hz), 1.51 (sept, 1 H, *J* = 6.6 Hz), 1.37–1.24 (m, 16 H), 1.15 (m, 2 H), and 0.86 (d, 6 H, *J* = 6.6 Hz).

**^13^C NMR (150 MHz, CDCl_3_):** 179.97, 39.05, 34.01, 29.93, 29.69, 29.63, 29.59, 29.42, 29.23, 29.05, 27.97, 27.41, 24.67, and 22.65.

**MS (ESI):** calculated for C_15_H_29_O_2_ [M-H]^−^ 241.2173, found 241.2.

### Effect of the DSF-Dependent Quorum-Sensing System on XSAC Production

The ability of the wild-type and DSF mutants to produce XSAC was measured by an anti-*Xanthomonas* activity assay (diameter of inhibition zone) and analyzed using the agar diffusion method as described below. Pathogenic strains of *Xanthomonas*, including *X. oryzae* pv. *oryzae* PXO99^A^, *X. oryzae* pv. *oryzae* RS105, *X. oryzae* pv. *oryzae* KACC10331, *X. campestris* pv. *campestris* 8004, and *X. axonopodis* pv. *glycines* 12-2, were individually incubated in NB liquid medium until the culture OD_600_ was approximately 1.0. *L. brunescens* OH23, and its derived mutants (Δ*rpfF*, Δ*rpfC*, and Δ*rpfG* mutants) were incubated in NB liquid medium or NB liquid medium supplemented with 2 μM *Lb*DSF until approximately 1.5–2.0 × 10^9^ CFU/ml (OD_600_ = approximately 1.5–2.0). The cultures of *L. brunescens* OH23, Δ*rpfF*, Δ*rpfC*, and Δ*rpfG* were centrifuged, and the supernatants were incubated at 85°C for 30 min. For each pathogenic strain of *Xanthomonas* (Table 1 and Supplementary Table S1), 100 ml of liquefied NB solid medium was incubated at 45°C for 30 min, mixed with 10^8^ cells, and then poured into plates. Then, 30 μl of supernatant from the cultures was spotted onto the selective plates. All plates were cultured at 28°C, and the zones of inhibition on the plates were photographed and compared after 2 days. XSAC production was confirmed by the diameter of the inhibition zone, and all of the data regarding the inhibition zone were subtracted from the diameter of the oxford cup. Three replicates were performed for each treatment, and the experiment was repeated three times.

### Pathogenicity Assays *in vivo*

*Oryza sativa* ssp. *indica* rice cultivars IR24 and *X. oryzae* pv. *oryzae* PXO99^A^ were used in the pathogenicity assay. *O. sativa* ssp. *indica* rice cultivars IR24 were grown under a 12-h light/dark cycle at 25°C with approximately 70% relative humidity for 2 months. The preparation of the supernatants from cultures of *L. brunescens* OH23, Δ*rpfF*, Δ*rpfC*, and Δ*rpfG* is described above. The leaves of IR24 plants were detached and dipped in the liquid culture of *X. oryzae* pv. *oryzae* PXO99^A^ at a concentration of approximately 0.5 × 10^9^ CFU/ml (OD_600_ = approximately 0.5) for 1 h. To determine the XSAC production and activity of wild-type OH23, Δ*rpfF*, Δ*rpfC*, and Δ*rpfG*, and chemically complemented strains, *X. oryzae* pv. *oryzae* PXO99^A^-infected rice leaves were treated with the respective supernatants every 24 h. For the negative control, *X. oryzae* pv. *oryzae* PXO99^A^-infected rice leaves were treated with NB medium. IR24 plants were grown in a glasshouse with the same conditions as above, and lesion lengths were measured and photographed at 7 dpi. Three replicates were performed for each treatment, and the experiment was repeated three times.

### Detection and Comparison of Colony Morphology in *L. brunescens*

Single colonies of wild-type OH23, Δ*rpfF*, Δ*rpfC*, and Δ*rpfG* strains were inoculated on NYG plates or NYG plates containing 5 μM *Lb*DSF for 3 days at 28°C. Then, the colony morphology of each strain was photographed and compared. Three replicates were used for each treatment, and the experiment was repeated three times.

### Observation of Surface Motility

The surface motility assay of *L. brunescens* wild-type OH23, Δ*rpfF*, Δ*rpfC*, and Δ*rpfG* strains was performed as previously described (Song et al., 2017). Briefly, NB semi-solid medium containing 0.3% agar was used for surface motility assays, and 2.5 μl of *L. brunescens* wild-type OH23 or the derived mutants (10^9^ CFU/ml, OD_600_ was approximately 1.0 for all strains) was spotted onto the surface of NB semi-solid medium plates or NB semi-solid medium plates containing 5 μM *Lb*DSF. The plates were incubated at 28°C for 4 days, and then the surface motility of each strain was photographed, measured, and compared. Three replicates were performed for each treatment, and the experiment was repeated three times.

### RNA Extraction, Reverse Transcription PCR, and Real-Time-PCR

*Lysobacter brunescens* OH23, Δ*rpfF*, Δ*rpfC*, and Δ*rpfG* mutants were each grown in 5 ml of NA medium to an OD_600_ of approximately 1.0. Three milliliters of cells was transferred into a sterile centrifuge tube and centrifuged for 3 min at 12,000 rpm. RNA was extracted from the strains using TRIzol solution (TaKaRa Biocompany) following the manufacturer’s instructions. For DNA removal and reverse transcription PCR, the PrimerScript RT Reagent Kit with the gDNA Eraser Kit (TaKaRa Biocompany) was used in this study. For the real-time PCR assay, a QuantStudio 6 Flex Real-Time PCR System (Thermo Fisher Scientific) was used to detect gene expression. The gene expression was calculated by the 2^−ΔΔ^*^CT^* method. The primers for real-time PCR are listed in Supplementary Table S2. Three replicates were performed for each treatment, and the experiment was repeated three times.

### Data Analysis

Statistical analyses were calculated using SPSS (Statistical Package, Version 21.0). The variables were subjected to Student’s *t*-test and tested for significance at *P* < 0.05 (^∗^), *P* < 0.01 (^∗∗^), *P* < 0.001 (^∗∗∗^), and *P* < 0.0001 (^∗∗∗∗^).

## Results and Discussion

### Identification of the Small Signaling Molecule *Lb*DSF in *L. brunescens*

**FIGURE 1:**
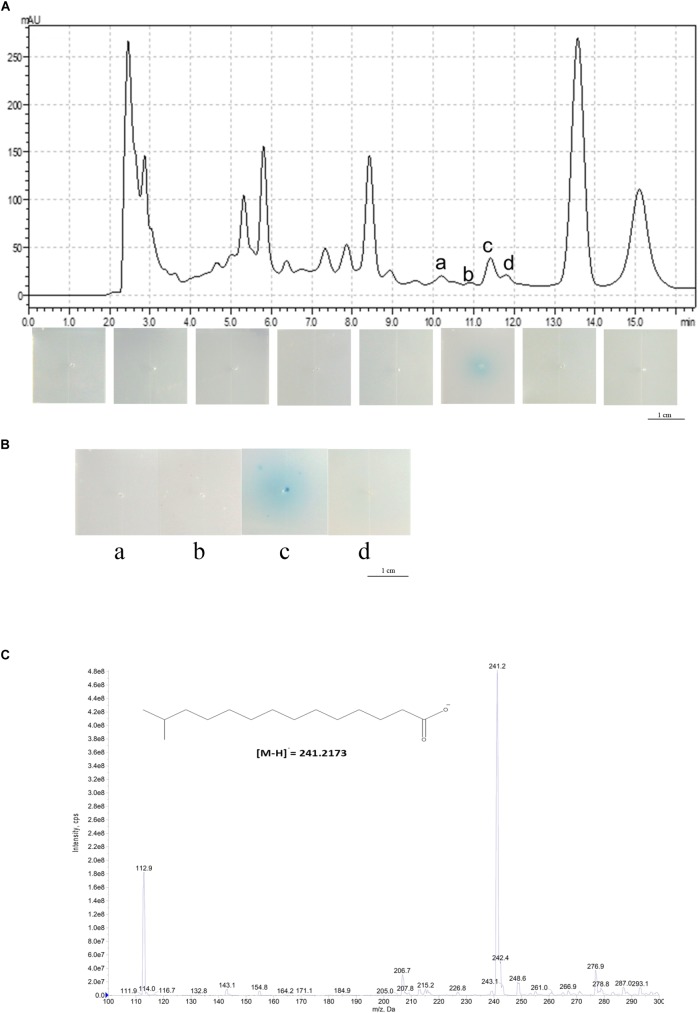
Structural determination of the DSF from *L. brunescens* OH23. **(A)** HPLC analysis of the *L. brunescens* crude supernatant. **(B)** Bioassay of the four purified HPLC fractions a, b, c, and d from 10 to 12 min. The blue halo indicates DSF activity. **(C)** Mass spectrometry analysis of *Le*DSF3 (calculated [M-H]^−^ = 241.2173 Da, observed 241.2 Da).

To identify the structure of the DSF signal in *L. brunescens* OH23, we harvested the *L*. *brunescens* culture from a 50-L fermenter system (CRJ-50D), collecting 25 L each time for a total of 50 L, and we extracted the crude DSF from the supernatant using the same method as previously described (Han et al., 2015). The methods of separation and identification of the DSF from OH23 were described previously (Wang et al., 2004). Briefly, we collected samples every 2 min from HPLC, concentrated every fraction by evaporation, and added 1 μg of every sample into the DSF bioassay plates containing *Xcc* FE58 and X-gluc. The formation of a blue halo in the plates indicated the induction ability of each fraction. The results showed that the samples from 10.01 to 12.00 min induced *gusA* expression (Figure 1A,B). The DSF activity from 10.01 to 12.00 min was 0.53 ± 0.20 units. Furthermore, P*_engXCA_*-*gusA* was minimally induced by the addition of the samples from 0.01–10.00 or 12.01–16.00 min, and blue halo zones were not observed. To further confirm these results, we purified 1 μg of compound from these four fractions and detected the induction abilities of each fraction using the DSF bioassay system. The results of this assay revealed that P*_engXCA_*-*gusA* activation was induced only by adding the purified compound from fraction c, which exhibited a DSF activity of 1.98 ± 0.69 units.

We also examined whether the compound from these fractions enhanced the anti-*Xanthomonas* activity in *L. brunescens* OH23. We collected, dried, and dissolved every fraction in DMSO. The supernatant from strain OH23 formed a zone of inhibition in the growth of *X. oryzae* pv. *oryzae* RS105 that was 2.15 ± 0.09 cm in diameter. The zone of inhibition increased to 2.77 ± 0.12 cm after the addition of 2.0 μM purified fraction c (Supplementary Figure S3). Moreover, the zone of inhibition did not significantly increase when the purified compounds from fraction a, fraction b, fraction d, or the DMSO control were added (Supplementary Figure S3). These results demonstrated that only the compounds from fraction c, collected at 11.5 min, enhanced the inhibition activities in strain OH23 (Supplementary Figure S3).

To identify the structure of the unidentified DSF compounds, we first collected approximately 2.6 mg of the compounds exhibiting the highest DSF activity (fraction c, 11.5 min) from 50 L of crude supernatant and resuspended it in chloroform for NMR analysis. After purification of the compounds from fraction c, mass spectrometry analysis of the purified compound revealed a main *m/z* fraction at 241.2 Da, which was consistent with the expected molecular weight of *Le*DSF3, [M-H]^−^ = 241.2173 Da (Figure 1C). In contrast with most DSFs, the structure of *Le*DSF3 consisted of a saturated aliphatic chain, which was formed by a tetradecanoic acid structure with a methyl group linked to carbon 13. To confirm the presence of *Le*DSF3, ^1^H and ^13^C NMR spectra were collected, and all signals were assigned (Supplementary Figures S2A,B). In agreement with the expected structure, no signal was detected between 7 and 5 ppm in the ^1^H NMR spectrum, indicating that the purified compound was not an unsaturated aliphatic chain. A doublet with a 6.6-Hz coupling constant appeared at 0.86 ppm in the ^1^H NMR spectrum, corresponding to the methyl groups. The protons in the alpha and beta position to the carboxylic acid were observed at 2.35 and 1.63 ppm as a triple and a quintuplet, respectively, whereas the proton of carbon **13** appeared at 1.51 ppm as a septuplet. ^13^C NMR revealed a signal at 179.97 ppm, corresponding to the carboxylic acid. Carbon **2** was detected at 34.01 ppm, whereas the methyl groups appeared at 22.65 ppm in the ^13^C NMR spectrum. In agreement with the ^1^H NMR spectrum, no signal was detected between 180 and 120 ppm in the ^13^C NMR spectrum, discarding the possibility of an unsaturated chain. Therefore, the primary compound from fraction c, named *Lb*DSF, was determined to be 13-methyltetradecanoic acid (Figure 1C), which was originally reported in *L. enzymogenes* (Han et al., 2015).

### Identification of the *rpf* Gene Cluster in *L. brunescens*

**FIGURE 2:**
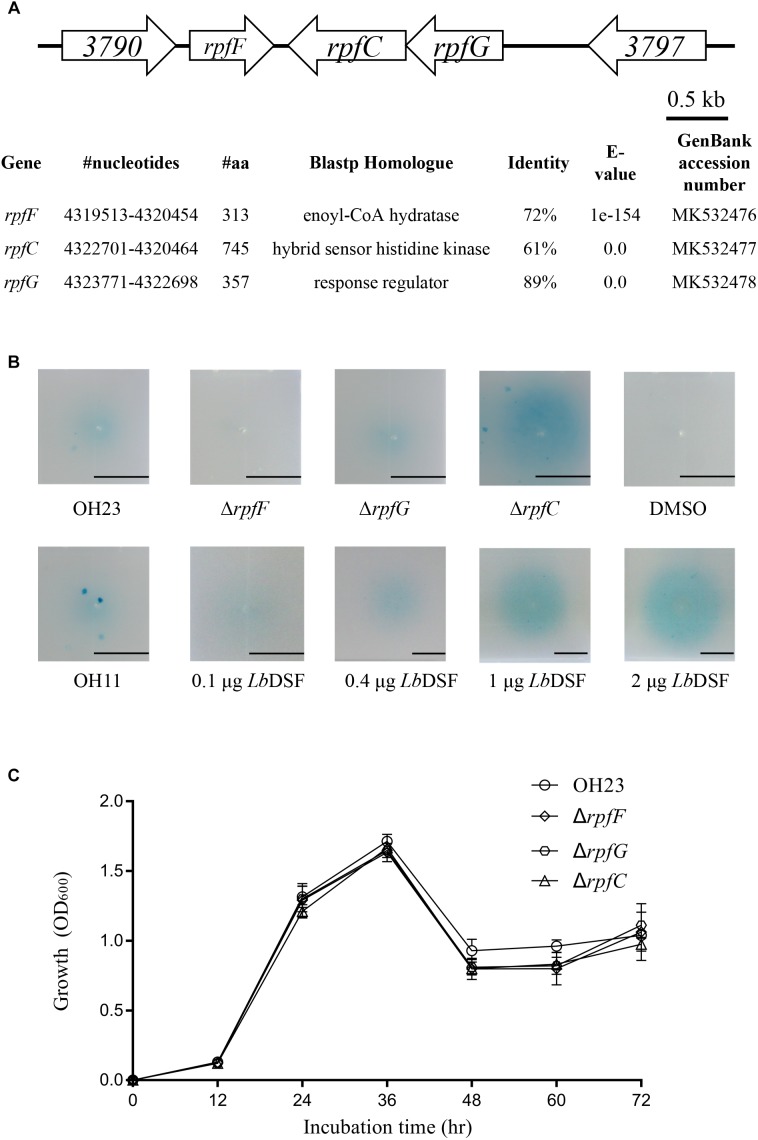
Location of the *rpf* genes in the genome of *L. brunescens* OH23 and the DSF activity of the different *L. brunescens* OH23, *rpf* mutants, or *L. enzymogenes* OH11. **(A)** BLASTp comparison between the proteins encoded by the *rpf* genes (*rpfF*, *rpfC*, and *rpfG*) of *L. brunescens* and *L. enzymogenes*. The protein similarity was compared using BioEdit7.0.9.0. **(B)** The DSF production ability of strain OH23 and its corresponding *rpf* mutants. **(C)** The growth of wild-type OH23 and its corresponding *rpf* mutants.

As mentioned above, DSF family signals have been reported in different *Xanthomonas* bacteria. As a result, we compared and sequenced the genome of OH23 to identify genes potentially related to the *Lb*DSF biosynthesis pathway. By blasting all *rpf* operon protein sequences^1^, we found that *rpf* proteins were highly conserved in *Lysobacter* spp. NCBI BLASTp analysis revealed that the Rpf proteins from *L. brunescens* had the highest homology to those of *L. enzymogenes* (taxid: 69), and the levels of protein identity were 72% (*rpfF*), 61% (*rpfC*), and 89% (*rpfG*). According to our BLASTp results, RpfF is an enoyl-CoA hydratase involved in the biosynthesis of DSF (Figure 2A). Accordingly, the DSF activity by wild-type OH23 was 0.88 ± 0.28 units, and the mutation of the *rpfF* gene abolished DSF production in *L. brunescens* (Figure 2B). RpfC is the hybrid histidine sensor kinase of the Rpf two-component regulatory system, which negatively regulates DSF production. Therefore, DSF accumulated in the Δ*rpfC* mutant (Figure 2A,B), and DSF production of Δ*rpfC* was 5.91 ± 2.29 units. RpfG is the response regulator of the Rpf two-component regulatory system and is responsible for signal transduction. Compared to wild-type OH23, DSF production was not altered in the Δ*rpfG* mutant (Figure 2A,B), and the DSF activity of the Δ*rpfG* was 0.63 ± 0.14 units.

Mutation of *rpfF* abolished the ability to produce DSFs in *X. campestris* pv *campestris*, which suggested that *rpfF* were essential for DSF production (Deng et al., 2011). In *X. oryzae* pv *oryzae*, the DSF production increased in the mutant of *rpfC*, and the DSF sensor kinase RpfC negatively regulated the biosynthesis of DSF (He et al., 2010; Deng et al., 2011). Taken together, these results indicate that the model for DSF production and signal transduction in *L. brunescens* is highly similar to that of *L. enzymogenes* and *X. oryzae* pv *oryzae* (He et al., 2010; Qian et al., 2013). In addition, the growth of wild-type OH23 and the *rpf* mutants was measured. The results demonstrated that the *rpf* mutants had growth similar to that of the wild-type strain under the tested conditions (Figure 2C).

### DSF-Dependent Quorum-Sensing System Positively Regulates Antibiotic Biosynthesis Through the Small Signaling Molecule *Lb*DSF and the RpfC/RpfG Two-Component System

**FIGURE 3:**
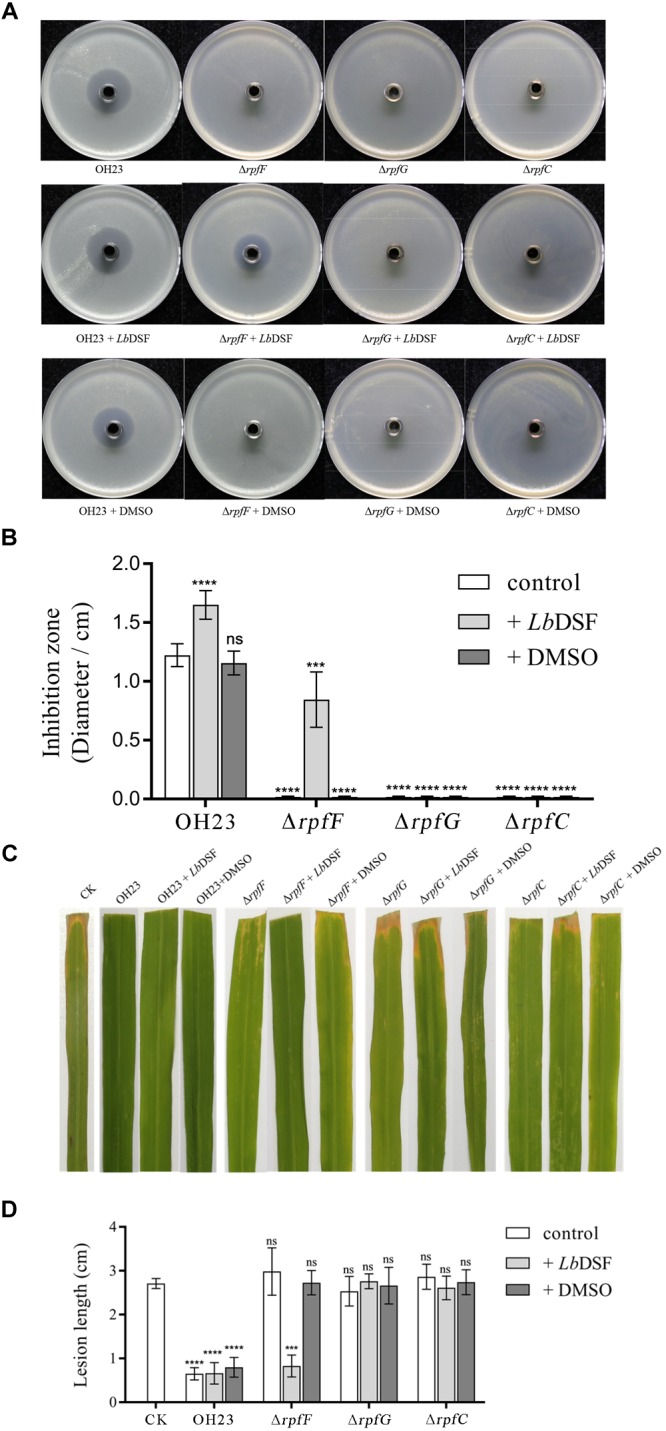
Anti-*Xanthomonas* activity of *L. brunescens* OH23 and its corresponding *rpf* mutants with or without *Lb*DSF addition. **(A)** Supernatants from *L. brunescens* OH23 or *rpf* mutant cultures with or without exogenous *Lb*DSF supplementation were tested for their antimicrobial activity against *X. oryzae* pv. *oryzae* PXO99^A^. **(B)** The analysis of the images of *X. oryzae* pv. *oryzae* PXO99^A^ growth inhibition zones shown in A. **(C)** Representative images of IR24 leaves infected with *X. oryzae* pv. *oryzae* PXO99^A^ and treated with test supernatants. CK is the control, which was treated with water every 24 h. Images were taken 7 days post-inoculation (dpi). **(D)** Analysis of the lesion lengths on IR24 rice leaves caused by *X. oryzae* pv. *oryzae* PXO99^A^ infection with or without treatment with test supernatants, as shown in **C**. Different numbers of star (^∗^) above the bars indicate a significant difference between the wild-type strain OH23 and mutant strains (ns: not sigificant, ^∗∗∗^*P* < 0.001; ^∗∗∗∗^*P* < 0.001, *t*-test).

In previous findings, the genus *Xanthomonas* and several Gram-negative bacteria used the DSF-dependent quorum-sensing system to mediate a diverse range of physiological activities related to virulence, motility, biofilm, and extracellular enzyme, and the DSF-dependent quorum-sensing system was also required for the biosynthesis of HSAF in *L. enzymogenes* (Qian et al., 2013; Guo et al., 2019). To investigate the function of the DSF-dependent quorum-sensing system in the biosynthesis of XSAC, we tested the anti-*Xanthomonas* abilities of the OH23 wild-type, Δ*rpfF*,Δ*rpfG*, Δ*rpfC*, and chemically complemented strains (mutants supplemented with *Lb*DSF). As shown in Figure 3A,B and Supplementary Figures S4–S7, wild-type OH23 inhibited the growth of *X. oryzae* pv. *oryzae* PXO99^A^ (*Xoo* PXO99^A^), *X. oryzae* pv. *oryzae* RS105 (*Xoo* RS105), *X. oryzae* pv. *oryzae* KACC 10331 (*Xoo* KACC 10331), *X. campestris* pv. *campestris* 8004 (Xcc 8004), and *X. axonopodis* pv. *glycines* 12-2 (*Xag* 12-2). The diameters of the inhibition zones were 1.22 ± 0.10 cm, 1.75 ± 0.15 cm, 1.95 ± 0.24 cm, 0.29 ± 0.09 cm, and 1.50 ± 0.14 cm, respectively. The Δ*rpfF*,Δ*rpfG*, and Δ*rpfC* mutants lost the ability to inhibit all tested *Xanthomonas* strains, demonstrating that these mutants were impaired in the biosynthesis of XSAC (Figure 3A and Supplementary Figures S4–S7). To determine the genes relevant to the XSAC biosynthesis, we analyzed the putative operon that related to the biosynthesis of XSAC (data not shown), and 8 genes of 13 genes were indispensable for the biosynthesis of XSAC (Supplementary Figures S8A,B). Furthermore, the q-PCR results shown that the expression of all 13 genes, the genes for XSAC biosynthesis, were dramatically reduced in the Δ*rpfF* mutant (Supplementary Figure S8C).

In *L. enzymogenes*, HSAF biosynthesis gene *pks-nrps* expression was reduced ∼10 times, and HSAF production was also dramatically reduced in the Δ*rpfF*_OH11_ mutant (Qian et al., 2013). Taken together, the DSF quorum-sensing XSAC biosynthesis regulation model in *L. brunescens* OH23 was similar to the DSF quorum-sensing HSAF biosynthesis regulation model in *L. enzymogenes* OH11, and the DSF quorum-sensing system positively regulated the biosynthesis of XSAC (Qian et al., 2013; Han et al., 2015).

To further address whether the small signaling molecule *Lb*DSF restored XSAC biosynthesis in the *rpf* mutants, we added *Lb*DSF (2 μM) to the mutant culture in liquid NB medium, incubated the culture at 28°C at 180 rpm, and then tested the anti-*Xanthomonas* activity of the supernatant. When *Lb*DSF was added into the cultures, the growth inhibitory ability of wild-type OH23 increased 35.01, 19.54, 8.97, 16.86, and 27.15% against *Xoo* PXO99^A^, *Xoo* RS105, *Xoo* KACC 10331, *Xcc* 8004, and *Xag* 12-2, respectively (Figure 3A and Supplementary Figures S4–S7). *Lb*DSF addition restored the anti-*Xanthomonas* activity in the Δ*rpfF*, and the diameter of the zones of inhibition were 0.84 ± 0.22 cm, 1.71 ± 0.16 cm, 0.36 ± 0.10 cm, 0.25 ± 0.11 cm, and 1.18 ± 0.16 cm against *Xoo* PXO99^A^, *Xoo* RS105, *Xoo* KACC 10331, *Xcc* 8004, and *Xag* 12-2, respectively (Figure 3A and Supplementary Figures S4–S7). Additionally, exogenous *Lb*DSF had no effect on the anti-*Xanthomonas* activity in Δ*rpfC* and Δ*rpfG* (Figure 3A). These results were consistent with the fact that the small signaling molecule *Lb*DSF is a transduction signal in the *L. brunescens rpf* system, where RpfF is involved in DSF biosynthesis, and RpfC/RpfG is the two-component system involved in signal transduction.

We then investigated whether all of the supernatants mentioned above had inhibitory activities *in vivo*. *Xoo* PXO99^A^-infected IR24 rice leaves were treated with the supernatants every 24 h, and lesion lengths were measured at 7 dpi. As shown in Figure 3C,D, the negative control was only treated with NB medium, and the lesion length was 2.92 ± 0.41 cm. However, the wild-type OH23 treatment group showed significantly decreased lesion lengths (0.70 ± 0.21 cm). The addition of exogenous *Lb*DSF or DMSO to the culture of wild-type OH23 was shown to significantly influence the lesion length relative to the negative control. For the Δ*rpfF* treatment group, exogenous *Lb*DSF restored the anti-*Xanthomonas* activity, and the lesion length was 0.83 ± 0.23 cm. However, both the Δ*rpfG* and Δ*rpfC* treatment groups exhibited similar lesion lengths as the negative control. The results from the *in vivo* plant assays were consistent with the results from the anti-*Xanthomonas* ability on plates.

In *L. enzymogenes*, *Le*DSF3 was 13-methyltetradecanoic acid, and acted as an extracellular signal to positively regulate the biosynthesis of HSAF; the two-component regulatory system RpfC/RpfG sensed and transduced *Le*DSF3; and the global regulator Clp was downstream of the *Le*DSF quorum-sensing system and also played a positive role in regulating the biosynthesis of HSAF and WAP-8294A2 (Han et al., 2015; Xu et al., 2016). To further investigate the function of *clp* in *L. brunescens* OH23, the Clp_OH11_ amino acid sequence was compared with the draft genome sequence of *L. brunescens* OH23, and Peg.2300 (Clp) shared 83% similarity to that of Clp_OH11_ at the amino acid level (Supplementary Figure S9A). Next, the anti-*Xanthomonas* abilities of Δ*clp* and its complementary strain were tested, and *clp* had no significant effect on biosynthesis of XSAC (Supplementary Figure S9B), which revealed that Clp was not involved in the regulation of XSAC biosynthesis. Taken together, these findings indicate that the structure of *Lb*DSF and the regulation of RpfC/RpfG *L. brunescens* were similar to the *Le*DSF quorum-sensing HSAF biosynthesis regulation model in *L. enzymogenes* and a potential novel transcription regulator in the DSF-dependent quorum-sensing system that regulated XSAC biosynthesis in *L. brunescens*.

### DSF-Dependent Quorum-Sensing System Affects Colony Morphology in *L. brunescens*

**FIGURE 4:**
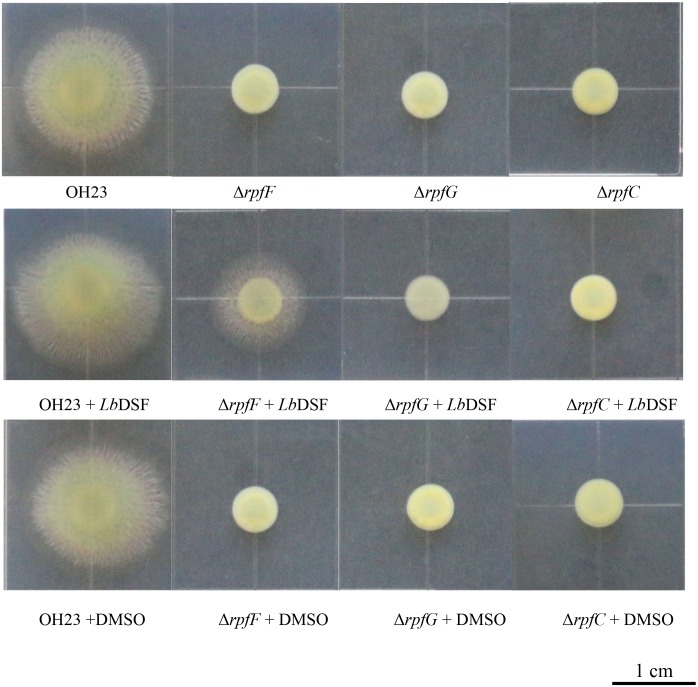
Colony morphology of wild-type OH23 and the *rpf* mutants on NYG plates with or without the exogenous addition of *Lb*DSF. Each strain was grown in NYG liquid medium until the OD_600_ was approximately 1.0. Then, 3 μl of each strain was inoculated onto the surface of NYG plates. The plates were incubated at 28°C for 4 days. For the *Lb*DSF treatment group, the strains were grown in NYG liquid medium containing 2 μM *Lb*DSF, and the NYG plates contained 5 μM *Lb*DSF. Each treatment was completed in triplicate, and the experiment was repeated three times.

The DSF quorum-sensing system was shown to influence colony morphology in *L. enzymogenes* (Qian et al., 2013). To investigate the function of the DSF-dependent quorum-sensing system in modulating colony morphology in *L. brunescens*, we tested the wild-type OH23 strain and the Δ*rpfF*, Δ*rpfC*, and Δ*rpfG* mutants. As shown in Figure 4, the wild-type OH23 displayed round colonies with lobular and spiculated boundaries on NYG plates with an average diameter size of 1.87 ± 0.05 cm. However, the Δ*rpfF*, Δ*rpfC*, and Δ*rpfG* mutants showed smooth colony appearance with an average diameter size of 0.64 ± 0.08 cm under the same growth conditions.

Exogenous *Lb*DSF (2 μM) restored the colony morphology in the Δ*rpfF* when compared to the wild-type OH23 with an average diameter size of 1.32 ± 0.08 cm, whereas the addition of exogenous *Lb*DSF did not influence the colony morphology of the Δ*rpfC* and Δ*rpfG* (Figure 4). These results indicate that the DSF-dependent quorum-sensing system is involved in the regulation of colony morphology in *L. brunescens*.

### DSF Signaling Controls Surface Motility Through Type IV Pili (T4P) in *L. brunescens*

The DSF-dependent quorum-sensing system was also shown to affect cell motility in *L. enzymogenes* (Qian et al., 2013). To investigate the function of the DSF-dependent quorum-sensing system in regulating motility in *L. brunescens*, we tested the motility of the wild-type OH23, Δ*rpfF*, Δ*rpfC*, and Δ*rpfG* strains. Since OH23, unlike *L. enzymogenes* OH11, does not exhibit twitching motility (data not shown), we analyzed the surface motility of wild-type OH23 and its derivative mutants on NB semi-solid (0.3% agar) motility medium plates for 4 days incubated at 28°C.

**FIGURE 5:**
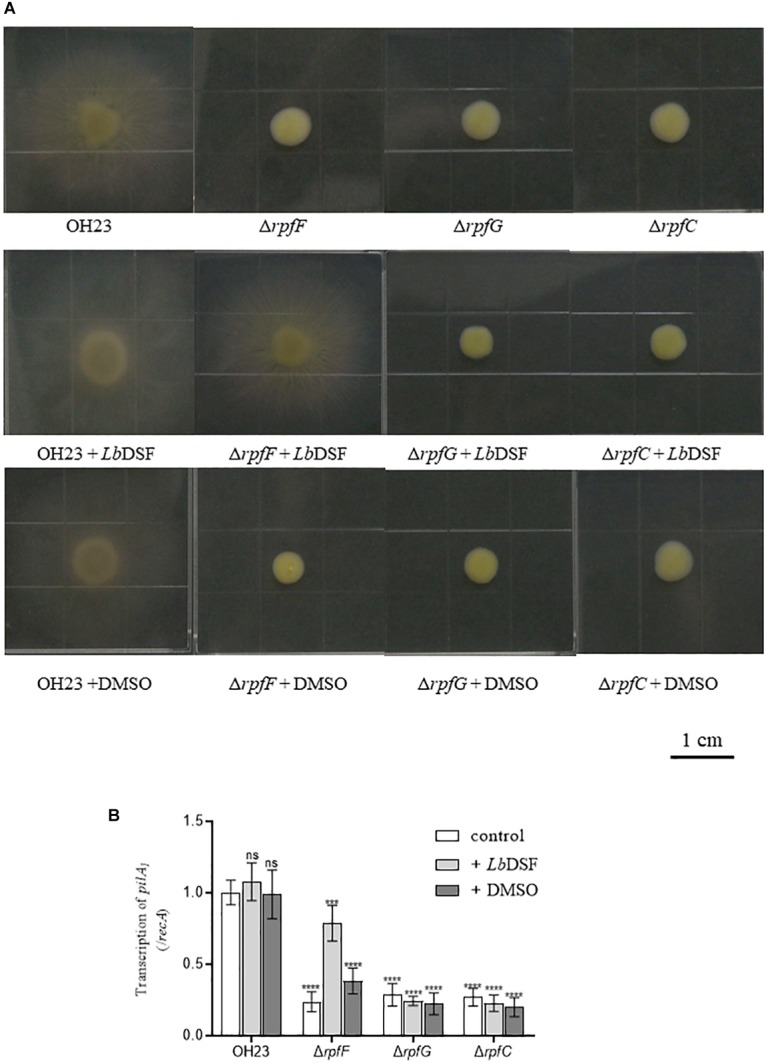
Surface motility of wild-type OH23 and the *rpf* mutants with or without the exogenous addition of *Lb*DSF. **(A)** Surface motility of wild-type OH23 and the *rpf* mutants with or without the exogenous addition of *Lb*DSF. Each strain was grown in liquid NB medium until the culture reached an OD_600_ of approximately 1.0. Then, 3 μl of strain was inoculated onto the surface of NB plates. The plates were inoculated at 28°C for 4 days. For the *Lb*DSF treatment group, the strains were grown in NYG liquid medium containing 2 μM *Lb*DSF. **(B)** *pilA*_1_ expression in wild-type OH23 and the *rpf* mutants with or without the exogenous addition of *Lb*DSF. Different numbers of star (^∗^) above the bars indicate a significant difference between the wild-type strain OH23 and mutant strains (ns: not sigificant, ^∗∗∗^*P* < 0.001; ^∗∗∗∗^*P* < 0.001, *t*-test).

As shown in Figure 5A, the wild-type OH23 strain was motile in motility medium plates with a typical circular dissemination pattern from the point of inoculation that was 2.78 ± 0.33 cm in size. However, the surface motility of Δ*rpfF*, Δ*rpfC*, or Δ*rpfG* was substantially reduced. These mutants only reached an average surface motility diameter of approximately 0.82 ± 0.11 cm in 4 days, indicating that the mutants had a 73.72% reduction in the diameter of their surface motility zone compared to the wild-type OH23. Exogenous *Lb*DSF (2 μM) restored surface motility in the Δ*rpfF* strain compared to the wild-type OH23 strain with an average surface motility diameter of 2.42 ± 0.10 cm. However, exogenous *Lb*DSF did not exhibit any effect on the surface motility in the Δ*rpfC* and Δ*rpfG* mutants. These results indicate that the DSF-dependent quorum-sensing system is involved in the regulation of surface motility in *L. brunescens.*

Type IV Pili has been shown to be important for surface motility in diverse bacteria. Since the *pilA* gene encodes the major pilin subunit of T4P (Mattick, 2002; Burdman et al., 2011; Wang et al., 2014), we measured *pilA_1_* expression in the wild-type OH23, Δ*rpfF*, Δ*rpfC*, and Δ*rpfG*. As shown in Figure 5B, *pilA_1_* expression was dramatically decreased in Δ*rpfF*, Δ*rpfC*, and Δ*rpfG*. The addition of exogenous *Lb*DSF (2 μM) partially restored *pilA_1_* expression in the Δ*rpfF* compared to wild-type OH23, while exogenous *Lb*DSF did not influence *pilA_1_* expression in the Δ*rpfC* and Δ*rpfG*. These results indicate that the DSF-dependent quorum-sensing system is involved in the regulation of *pilA_1_* expression in *L. brunescens*.

The DSF-dependent quorum-sensing system in the *Xanthomonas* genus differs substantially between species and has been shown to positively regulate virulence, biofilm formation, EPS biosynthesis, and adaption. Moreover, specific DSF molecules were related to specific antibiotic HSAF biosynthesis (He et al., 2010; Han et al., 2015). The Rpf system was shown to be activated by adding specific DSF molecules, and EPS production, extracellular xylanase activity, or antibiotic HSAF biosynthesis were restored (He et al., 2010; Han et al., 2015).

## Conclusion

**FIGURE 6:**
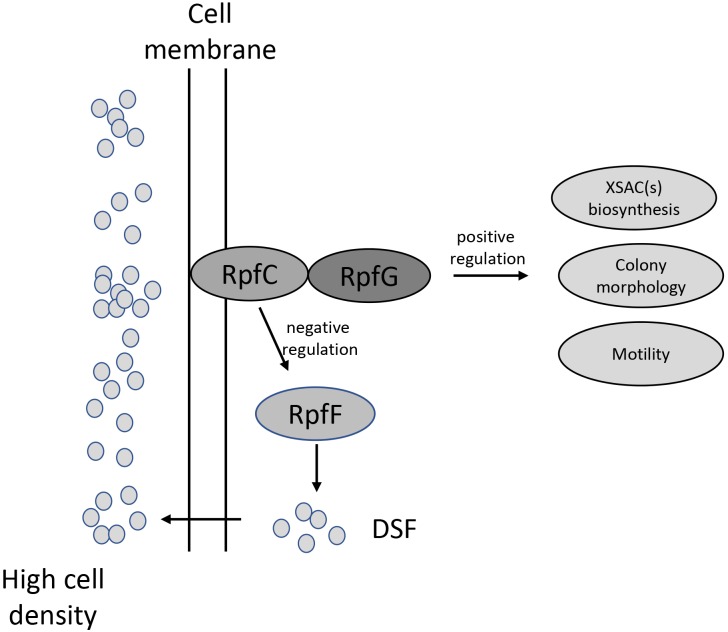
Schematic model of the DSF quorum-sensing system in *L. brunescens*. *Lb*DSF biosynthesis was found to be linked to RpfF (enoyl-CoA hydratase) and was dependent on the two-component regulatory system RpfC (hybrid sensor histidine kinase)/RpfG (response regulator). *Lb*DSF positively regulates XSAC biosynthesis, surface motility, and cell morphology.

In this study, we report the role of a quorum-sensing system in the production of a novel XSAC in the ubiquitous environmental bacterium *L. brunescens* (Figure 6). Our data revealed that *L. brunescens* OH23 uses a DSF-dependent quorum-sensing molecule to regulate XSAC production. We characterized this DSF compound as 13-methyltetradecanoic acid. This extracellular signal is produced by RpfF and transduced by the RpfC/RpfG two-component regulatory system. This molecule also regulates surface motility and colony morphology. Our findings will be useful in applied genetics and molecular biotechnology, thereby facilitating the improvement of antibiotic production in *Lysobacter* spp., which can potentially be used in the agricultural industry. Furthermore, the tight control that our identified DSF molecule exerts on XSAC expression suggests that *Lysobacter* spp. may produce other quorum-sensing signals that can induce the production of additional novel bioactive compounds.

## Data Availability

The datasets generated for this study can be found in Genbank, MK532476, MK532477, and MK532478.

## Author Contributions

JL, RZ, PL, TJ, YJ, and YZ conducted the experiments. FL designed and conducted the experiments. JL, PL, and FL contributed to the writing of the manuscript. FL revised the manuscript.

## Conflict of Interest Statement

The authors declare that the research was conducted in the absence of any commercial or financial relationships that could be construed as a potential conflict of interest.

## Footnotes

1 https://blast.ncbi.nlm.nih.gov/Blast.cgi

## References

[B1] Abisado R. G. Benomar S. Klaus J. R. Dandekar A. A. Chandler J. R. (2018). Bacterial quorum sensing and microbial community interactions. *mBio* 9:e02331-17. 10.1128/mBio.02331-17 29789364 PMC5964356

[B2] Atlas R. M. (1997). Handbook of microbiological media. *Q. Rev. Biol.* 2 364–365.

[B3] Barber C. E. Tang J. L. Feng J. X. Pan M. Q. Wilson T. J. Slater H. (1997). A novel regulatory system required for pathogenicity of *Xanthomonas campestris* is mediated by a small diffusible signal molecule. *Mol. Microbiol.* 24 555–566. 10.1046/j.1365-2958.1997.3721736.x 9179849

[B4] Burdman S. Bahar O. Parker J. K. De La Fuente L. (2011). Involvement of type IV pili in pathogenicity of plant pathogenic bacteria. *Genes* 2 706–735. 10.3390/genes2040706 24710288 PMC3927602

[B5] Christensen P. Cook F. D. (1978). Lysobacter, a new genus of nonfruiting, gliding bacteria with a high base ratio. *Int. J. Syst. Bacteriol.* 28 367–393. 10.1099/00207713-28-3-367

[B6] Deng Y. Y. Wu J. E. Tao F. Zhang L. H. (2011). Listening to a new language: DSF-based quorum sensing in gram-negative bacteria. *Chem. Rev.* 111 160–173. 10.1021/cr100354f 21166386

[B7] Guo W. Gao J. Chen Q. Ma B. Fang Y. Liu X. (2019). Crp-like protein (Clp) plays both positive and negative roles in regulating the pathogenicity of bacterial pustule pathogen *Xanthomonas axonopodis* pv. glycines. *Phytopathology* 10.1094/PHYTO-07-18-0225-R [Epub ahead of print]. 30730787

[B8] Han Y. Wang Y. Tombosa S. Wright S. Huffman J. Yuen G. (2015). Identification of a small molecule signaling factor that regulates the biosynthesis of the antifungal polycyclic tetramate macrolactam HSAF in *Lysobacter enzymogenes*. *Appl. Microbiol. Biotechnol.* 99 801–811. 10.1007/s00253-014-6120-x 25301587 PMC4308512

[B9] Harada S. Tsubotani S. Hida T. Ono H. Okazaki H. (1986). Structure of lactivicin, an antibiotic having a new nucleus and similar biological activities to β-lactam antibiotics. *Tetrahedron. Lett.* 27 6229–6232. 10.1016/s0040-4039(00)85439-8

[B10] Harada S. Tsubotani S. Ono H. Okazaki H. (1984). Cephabacins, new cephem antibiotics of bacterial origin. *J. Antibiot.* 37 1536–1545. 10.7164/antibiotics.37.1536 6526723

[B11] Hashizume H. Igarashi M. Hattori S. Hori M. Hamada M. Takeuchi T. (2001). Tripropeptins, novel antimicrobial agents produced by *Lysobacter* sp. I. taxonomy, isolation and biological activities. *J. Antibiot.* 54 1054–1059. 10.7164/antibiotics.54.1054 11858660

[B12] He Y. W. Wu J. E. Cha J. S. Zhang L. H. (2010). Rice bacterial blight pathogen *Xanthomonas oryzae* pv. oryzae produces multiple DSF-family signals in regulation of virulence factor production. *BMC Microbiol.* 10:187. 10.1186/1471-2180-10-187 20615263 PMC2909994

[B13] Holly S. Ariel A. M. Christine E. B. Michael J. D. Dow J. M. (2000). A two-component system involving an HD-GYP domain protein links cell-cell signalling to pathogenicity gene expression in *Xanthomonas campestris*. *Mol. Microbiol.* 38 986–1003. 10.1046/j.1365-2958.2000.02196.x 11123673

[B14] Kato A. Nakaya S. Ohashi Y. Hirata H. (1997). WAP-8294A(2), a novel anti-MRSA antibiotic produced by *Lysobacter* sp. *J. Am. Chem. Soc.* 119 6680–6681. 10.1021/ja970895o

[B15] Li S. J. Calvo A. M. Yuen G. Y. Du L. C. Harris S. D. (2009). Induction of cell wall thickening by the antifungal compound dihydromaltophilin disrupts fungal growth and is mediated by sphingolipid biosynthesis. *J. Eukaryot. Microbiol.* 56 182–187. 10.1111/j.1550-7408.2008.00384.x 21462551

[B16] Lou L. L. Qian G. L. Xie Y. X. Hang J. Chen H. Zaleta-Rivera K. (2011). Biosynthesis of HSAF, a tetramic acid-containing macrolactam from *Lysobacter enzymogenes*. *J. Am. Chem. Soc.* 133 643–645. 10.1021/ja105732c 21171605 PMC3078565

[B17] Maddocks S. E. Oyston P. C. F. (2008). Structure and function of the LysR-type transcriptional regulator (LTTR) family proteins. *Microbiol.* 154 3609–3623. 10.1099/mic.0.2008/022772-0 19047729

[B18] Mattick J. S. (2002). Type IV pili and twitching motility. *Ann. Rev. Microbiol.* 56 289–314. 10.1146/annurev.micro.56.012302.160938 12142488

[B19] Meyers E. Cooper R. Dean L. Johnson J. H. Slusarchyk D. S. Trejo W. H. (1985). Catacandins, novel anticandidal antibiotics of bacterial origin. *J. Antibiot.* 38 1642–1648. 10.7164/antibiotics.38.1642 4093330

[B20] Ono H. Nozaki Y. Katayama N. Okazaki H. (1984). Cephabacins, new cephem antibiotics of bacterial origin. *J. Antibiot.* 37 1528–1535. 10.7164/antibiotics.37.1528 6526722

[B21] O’Sullivan J. Mccullough J. E. Tymiak A. A. Kirsch D. R. Trejo W. H. Principe P. A. (1988). Lysobactin, a novel antibacterial agent produced by *Lysobacter* sp. I taxonomy, isolation and partial characterization. *J. Antibiot.* 41 1740–1744. 10.7164/antibiotics.41.1740 3209465

[B22] Qian G. L. Wang Y. L. Liu Y. R. Xu F. He Y. W. Du L. (2013). *Lysobacter enzymogenes* uses two distinct cell–cell signaling systems for differential regulation of secondary-metabolite biosynthesis and colony morphology. *Appl. Environ. Microbiol.* 79 6604–6616. 10.1128/AEM.01841-13 23974132 PMC3811492

[B23] Quandt J. Hynes M. F. (1993). Versatile suicide vectors which allow direct selection for gene replacement in gram-negative bacteria. *Gene* 127 15–21. 10.1016/0378-1119(93)90611-6 8486283

[B24] Robert P. R. Dow J. M. (2011). Communication with a growing family: diffusible signal factor (DSF) signaling in bacteria. *Trends Microbiol.* 19 145–152. 10.1016/j.tim.2010.12.003 21227698

[B25] Steven L. S. Daniel D. S. Michael C. S. Adam M. P. Pablo D. R. Seiji T. (2008). Genome sequence and rapid evolution of the rice pathogen *Xanthomonas oryzae* pv. oryzae PXO99A. *BMC Genomics* 9:204. 10.1186/1471-2164-9-204 18452608 PMC2432079

[B26] Sambrook J. (2001). *Molecular Cloning, a Laboratory Manual*. New York, NY: Hamlet.

[B27] Song Z. W. Zhao Y. C. Qian G. L. Odhiambo B. O. Liu F. (2017). Novel insights into the regulatory roles of gene hshB in *Xanthomonas* oryzae pv. oryzicola. *Res. Microbiol.* 168 165–173. 10.1016/j.resmic.2016.10.007 27810475

[B28] Van Houdt R. Moons P. Aertsen A. Jansen A. Vanoirbeek K. Daykin M. (2007). Characterization of a luxI/luxR-type quorum sensing system and *N*-acyl-homoserine lactone-dependent regulation of exo-enzyme and antibacterial component production in Serratia plymuthica RVH1. *Res. Microbiol.* 158 150–158. 10.1016/j.resmic.2006.11.008 17258895

[B29] Wang L. H. He Y. Gao Y. Wu J. E. Dong Y. H. He C. (2004). A bacterial cell–cell communication signal with cross-kingdom structural analogues. *Mol. Microbiol.* 51 903–912. 10.1046/j.1365-2958.2003.03883.x 14731288

[B30] Wang Y. Qian G. L. Liu F. Q. Shen Y. Du L. (2013). Facile method for site-specific gene integration in *Lysobacter enzymogenes* for yield improvement of the anti-MRSA antibiotics WAP-8294A and the antifungal antibiotic HSAF. *ACS Synth. Biol.* 2 670–678. 10.1021/sb4000806 23937053 PMC3830728

[B31] Wang Y. S. Zhao Y. X. Zhang J. Zhao Y. Shen Y. Su Z. (2014). Transcriptomic analysis reveals new regulatory roles of Clp signaling in secondary metabolite biosynthesis and surface motility in *Lysobacter enzymogenes* OH11. *Appl. Microbiol. Biotechnol.* 98 9009–9020. 10.1007/s00253-014-6072-1 25236801 PMC4207120

[B32] Xie Y. Stephen W. Shen Y. M. Du L. C. (2012). Bioactive natural products from Lysobacter. *Nat. Prod. Rep.* 29 1277–1287. 10.1039/c2np20064c 22898908 PMC3468324

[B33] Xu G. Shi X. F. Wang R. P. Xu H. Y. Du L. C. Chou S.-H. (2016). Insights into the distinct cooperation between the transcription factor Clp and LeDSF signaling in the regulation of antifungal factors in *Lysobacter* enzymogenes OH11. *Biol. Control* 120 52–58. 10.1016/j.biocontrol.2016.08.006

[B34] Zhang W. Li Y. Qian G. L. Wang Y. Chen H. Li Y. Z. (2011). Identification and characterization of the anti-methicillin-resistant *Staphylococcus aureus* WAP-8294A2 biosynthetic gene cluster from *Lysobacter* enzymogenes OH 11. *Antimicrob. Agents Chemother.* 55 5581–5589. 10.1128/aac.05370-11 21930890 PMC3232812

[B35] Zhao Y. Y. Qian G. L. Ye Y. H. Wright S. Chen H. Shen Y. (2016). Heterocyclic aromatic N-oxidation in the biosynthesis of phenazine antibiotics from *Lysobacter* antibioticus. *Org. Lett.* 18 2495–2498. 10.1021/acs.orglett.6b01089 27145204

