# Supplementary material for: LbDSF, the Lysobacter brunescens Quorum-Sensing System Diffusible Signaling Factor, Regulates Anti- Xanthomonas XSAC Biosynthesis, Colony Morphology, and Surface Motility
Source: Front Microbiol. 2019 Jun 18;10:1230. doi: 10.3389/fmicb.2019.01230 (PMC6591275; doi:10.3389/fmicb.2019.01230)
Supplement: Supplementary file 1 [file Data_Sheet_1.docx]

**
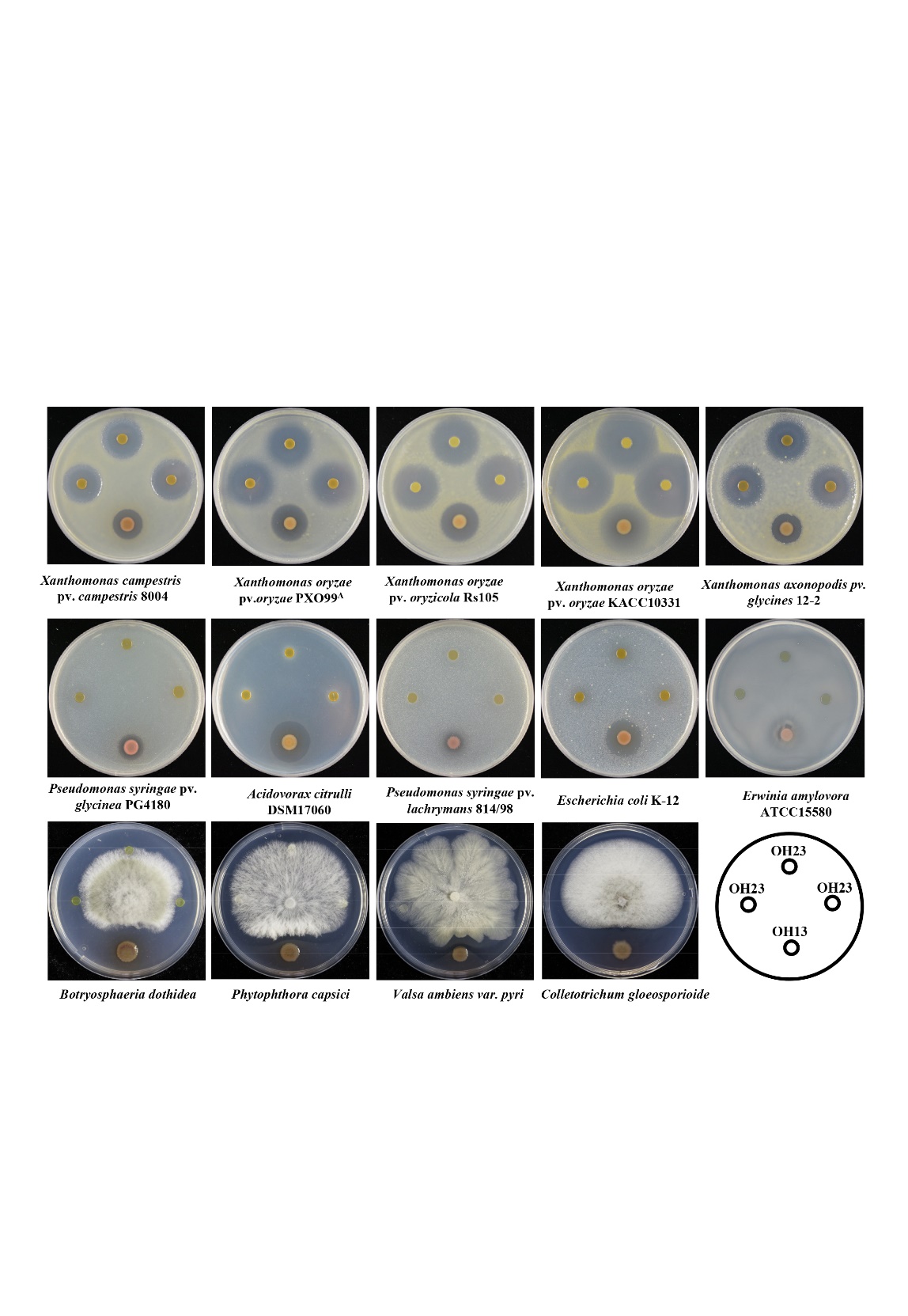
**

**Figure S1**. Growth-inhibitory activity of *L. brunescens* OH23 against the plant pathogen *Xanthomonas* spp. *X. campestris* pv. *campestris* 8004, *X. oryzae* pv. *oryzae* PXO99^A^, *X. oryzae* pv. *oryzicola* Rs105, *X. oryzae* pv. *oryzae* KACC10331, *X. axonopodis* pv. *glycines* 12-2, *P. syringae* pv. *glycinea* PG4180, *A. citrulli* DSM17060, *P. syringae* pv. *lachrymans* 814/98, *E. coli* K-12, *E. amylovora* ATCC15580, *B. dothidea*, *P. capsica*, *V. ambiens* var. Pyri, and *C. gloeosporioide* was used to detect the growth inhibitory activity of *L. brunescens* OH23. *L. antibioticus* OH13 was used as a control.

**
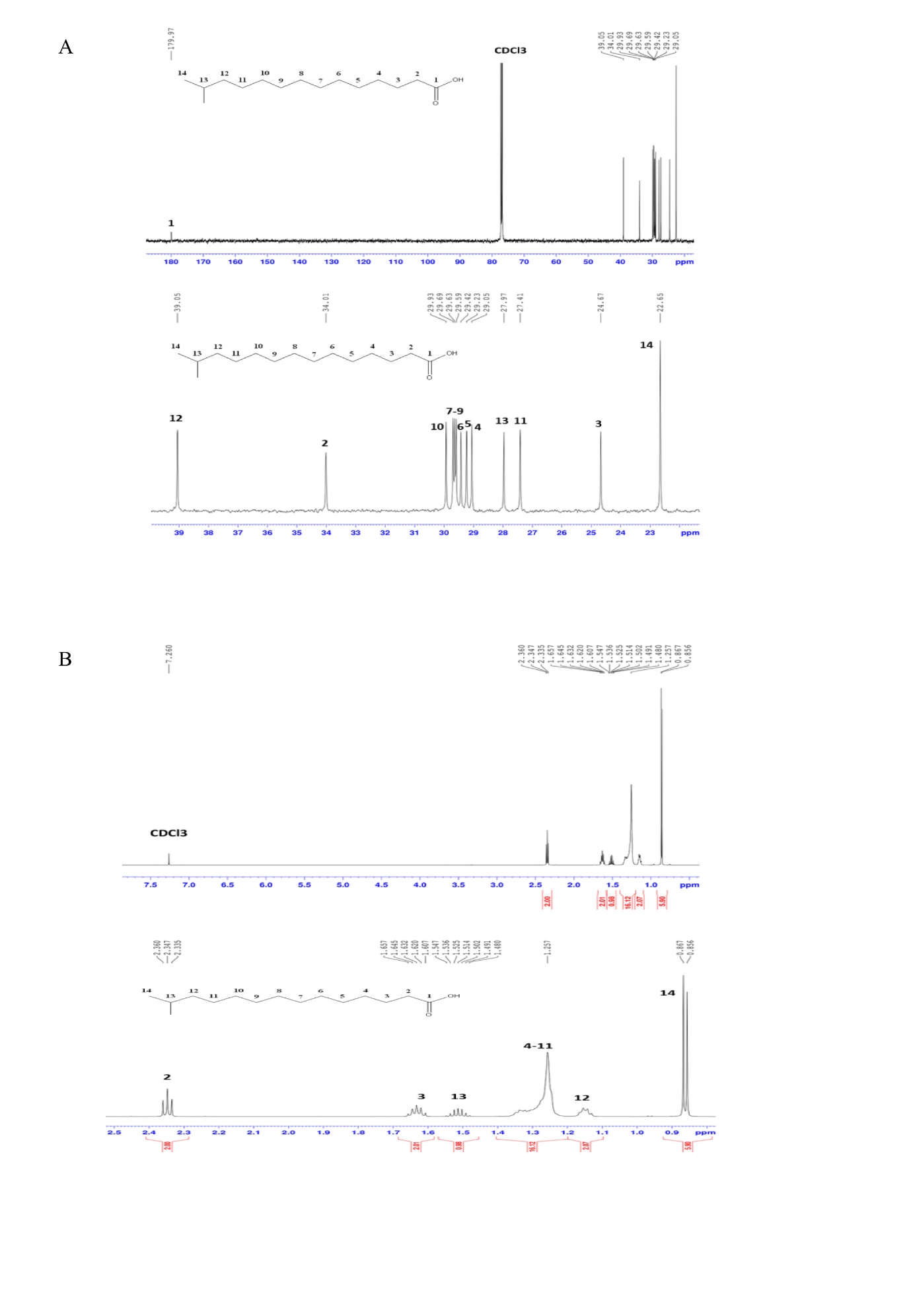
**

**Figure S2**. ^13^C NMR spectrum and ^1^H NMR spectrum analysis for *Lb*DSF. (A) The ^13^C NMR spectrum for *Lb*DSF. (B) The ^1^H NMR spectrum of *Lb*DSF.

**
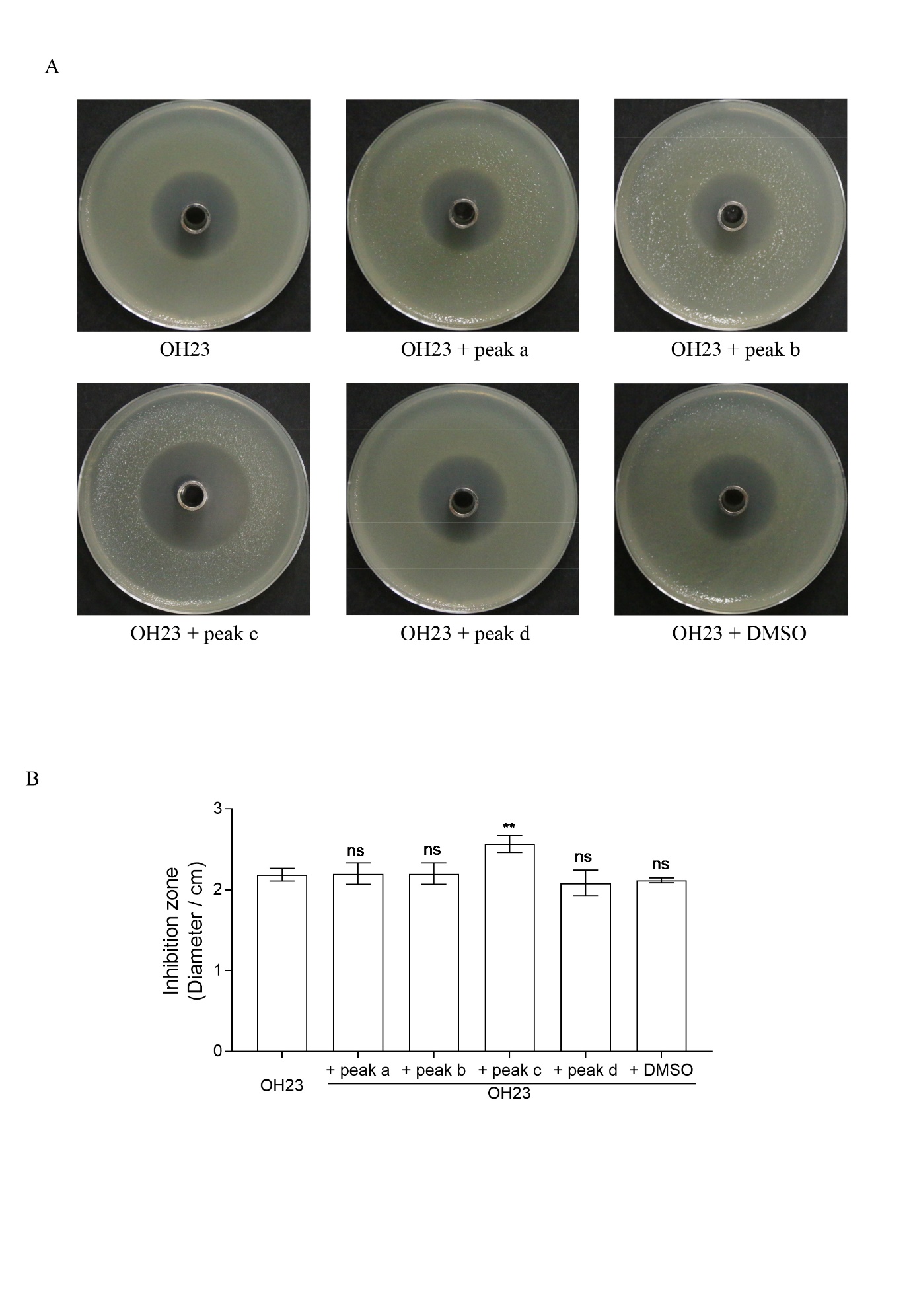
**

**Figure S3**. Growth-inhibitory activity of *L. brunescens* OH23 against the plant pathogen *X. oryzae* pv. *oryzicola* Rs105 was determined using HPLC-purified compounds from *L. brunescens* culture supernatants. The four compounds were collected from 10.01 to 12.00 min. They were evaporated to dryness under reduced pressure, and 1 μg of each compound was dissolved in 30 μl of methanol to inhibit *Xanthomonas* spp. activity.

**
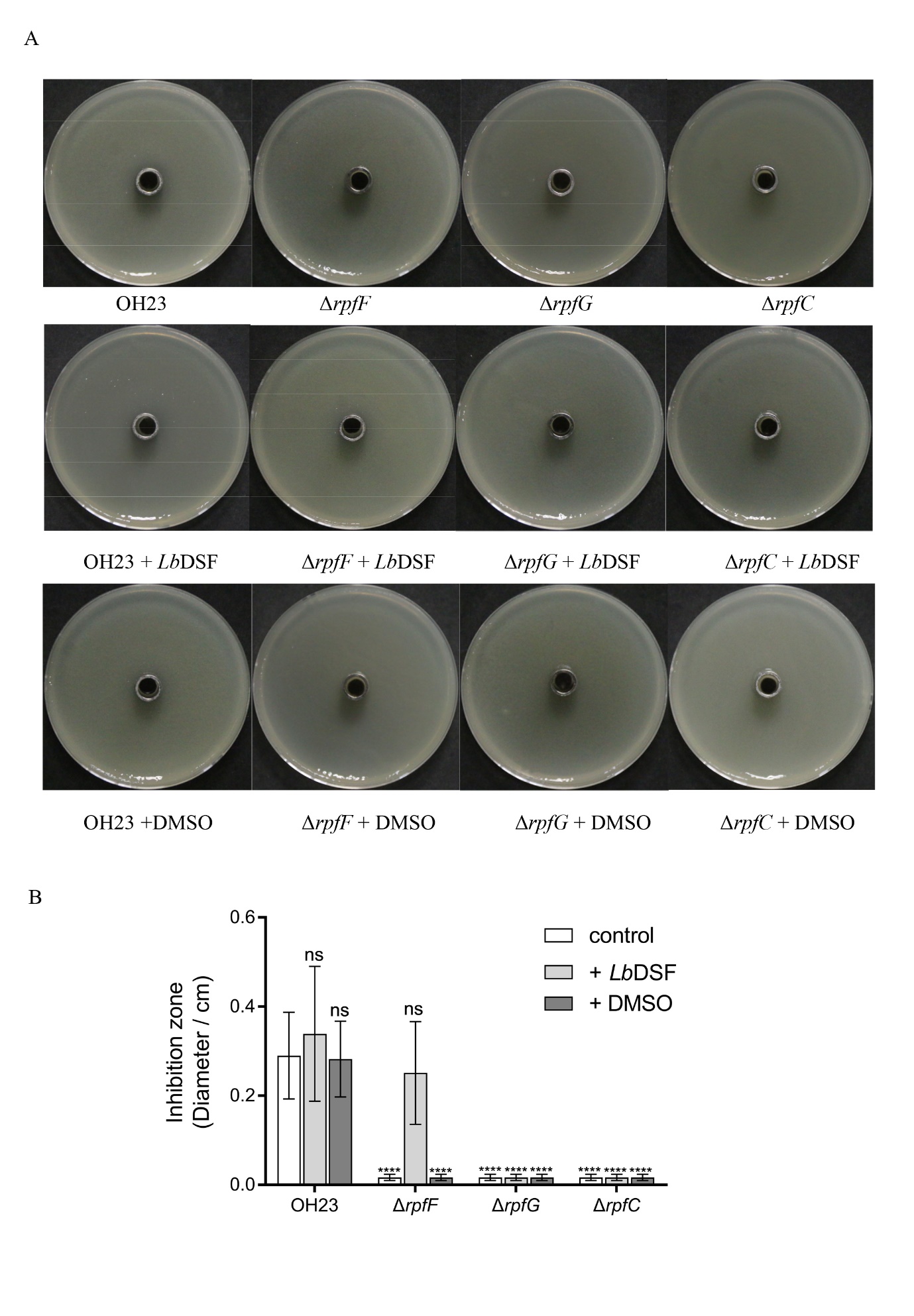
**

**Figure S4**. Determination of the ability of wild-type OH23 or the Δ*rpfF*, Δ*rpfC*, and Δ*rpfG* mutants to inhibit the growth of the plant pathogen *X. campestris* pv. *campestris* 8004 with or without 2 μM *Lb*DSF.

**
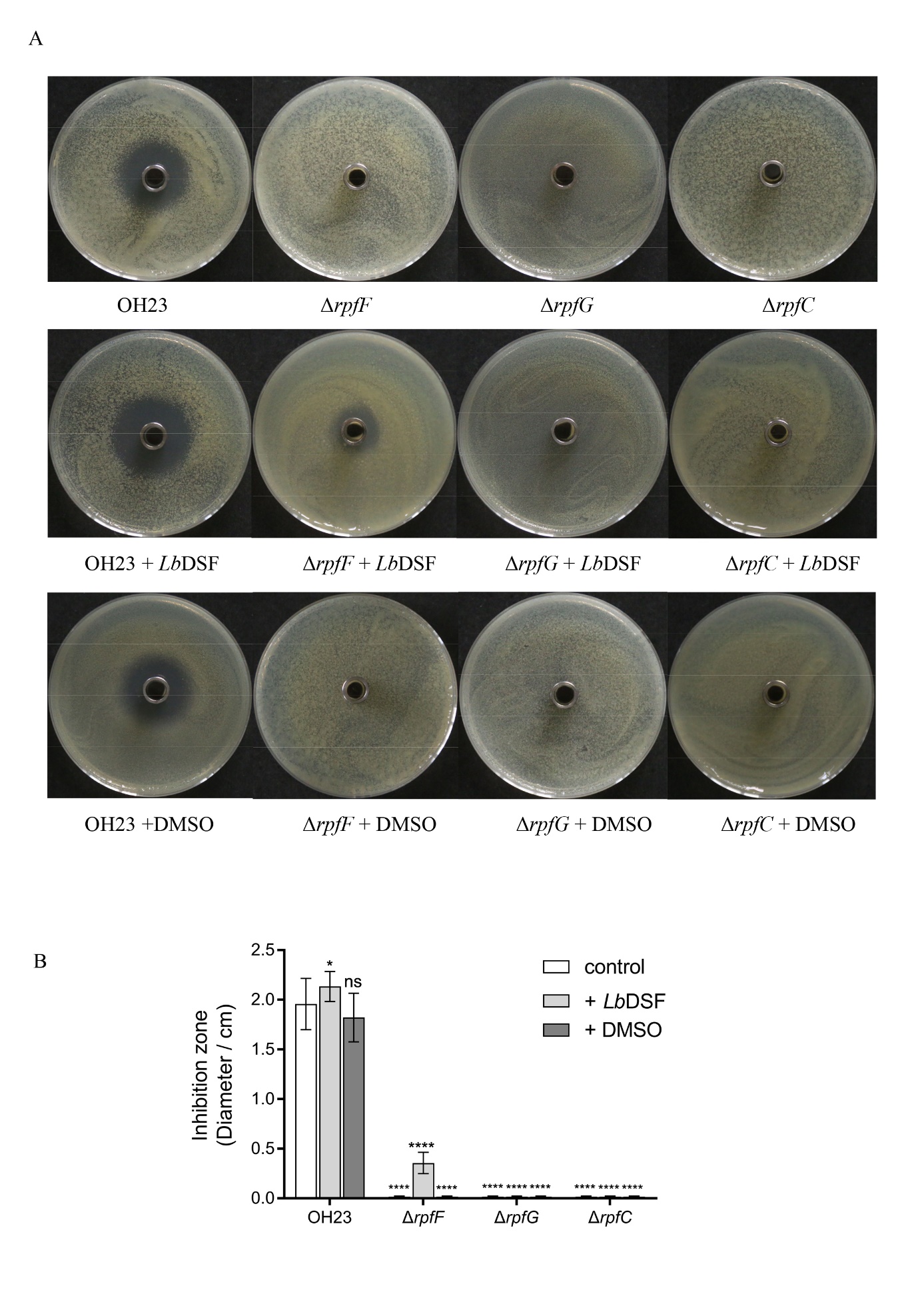
**

**Figure S5**. Determination of the ability of wild-type OH23 or the Δ*rpfF*, Δ*rpfC*, and Δ*rpfG* mutants to inhibit the growth of the plant pathogen *X. oryzae* pv. *oryzae* KACC10331 with or without 2 μM *Lb*DSF.

**
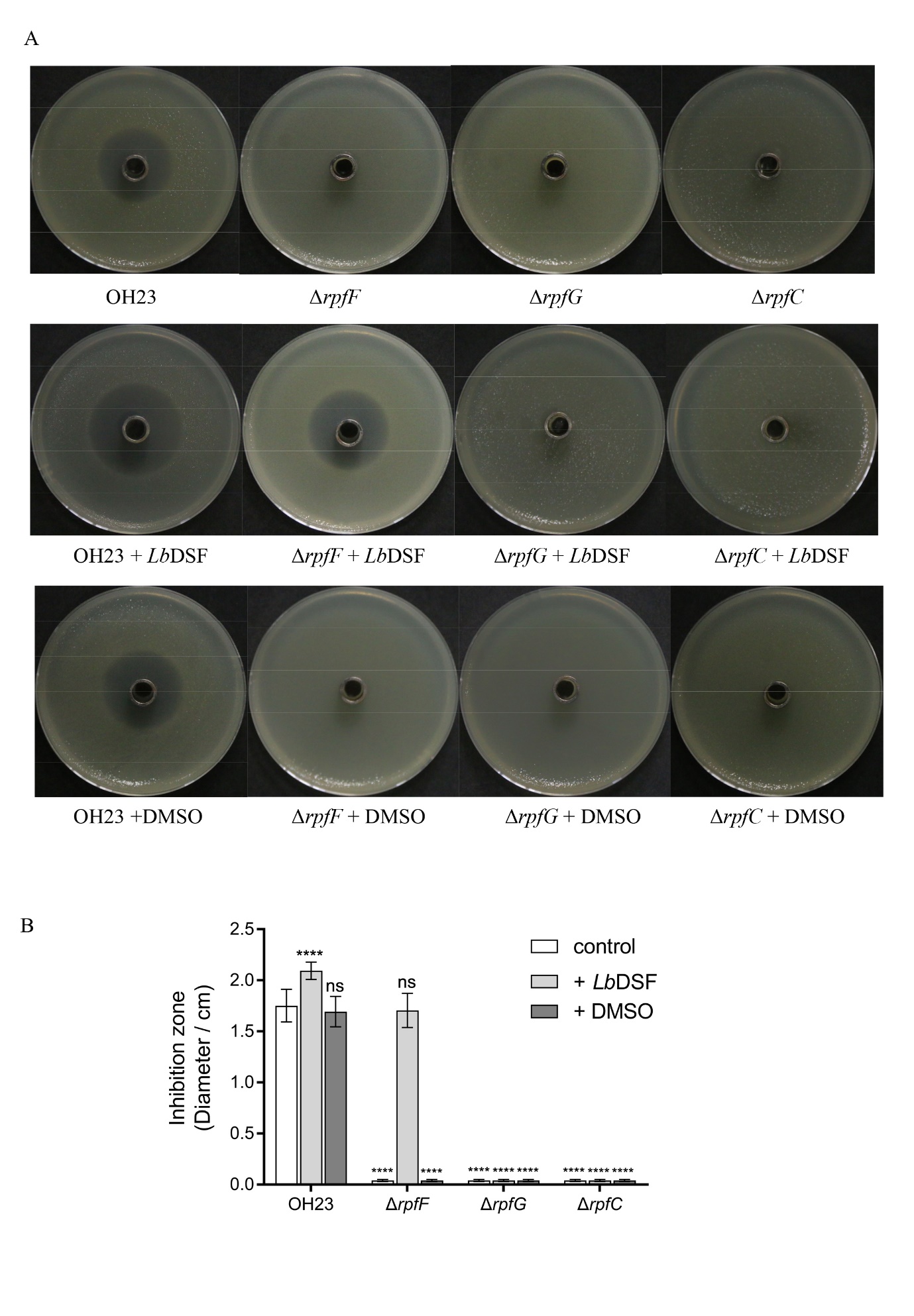
**

**Figure S6**. Determination of the ability of wild-type OH23 or the Δ*rpfF*, Δ*rpfC*, and Δ*rpfG* mutants to inhibit the growth of the plant pathogen *X. oryzae* pv. *oryzae* RS105 with or without 2 μM *Lb*DSF.


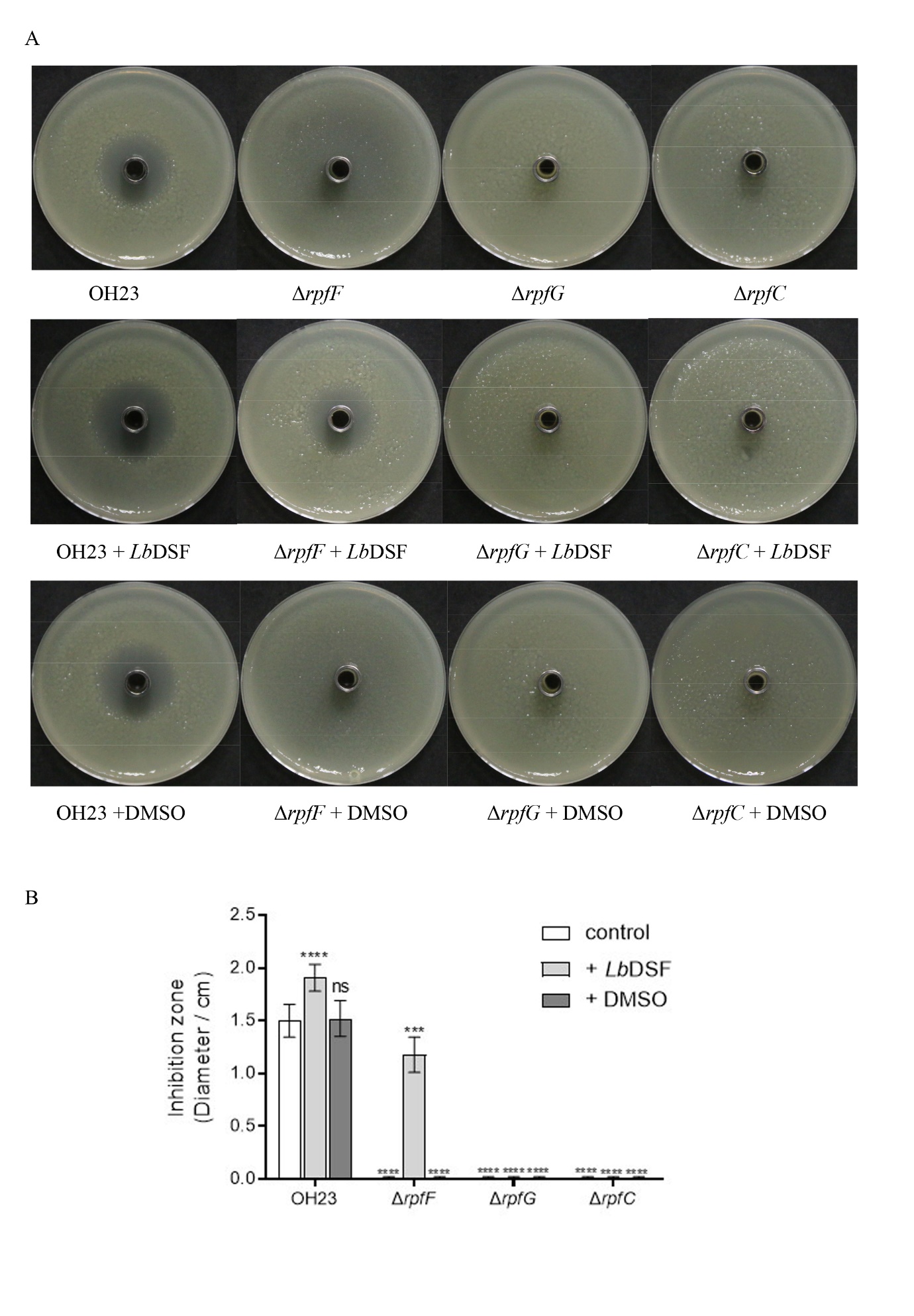


**Figure S7**. Determination of the ability of wild-type OH23 or the Δ*rpfF*, Δ*rpfC*, and Δ*rpfG* mutants to inhibit the growth of the plant pathogen *X. axonopodis* pv. *glycines* 12-2 with or without 2 μM *Lb*DSF.


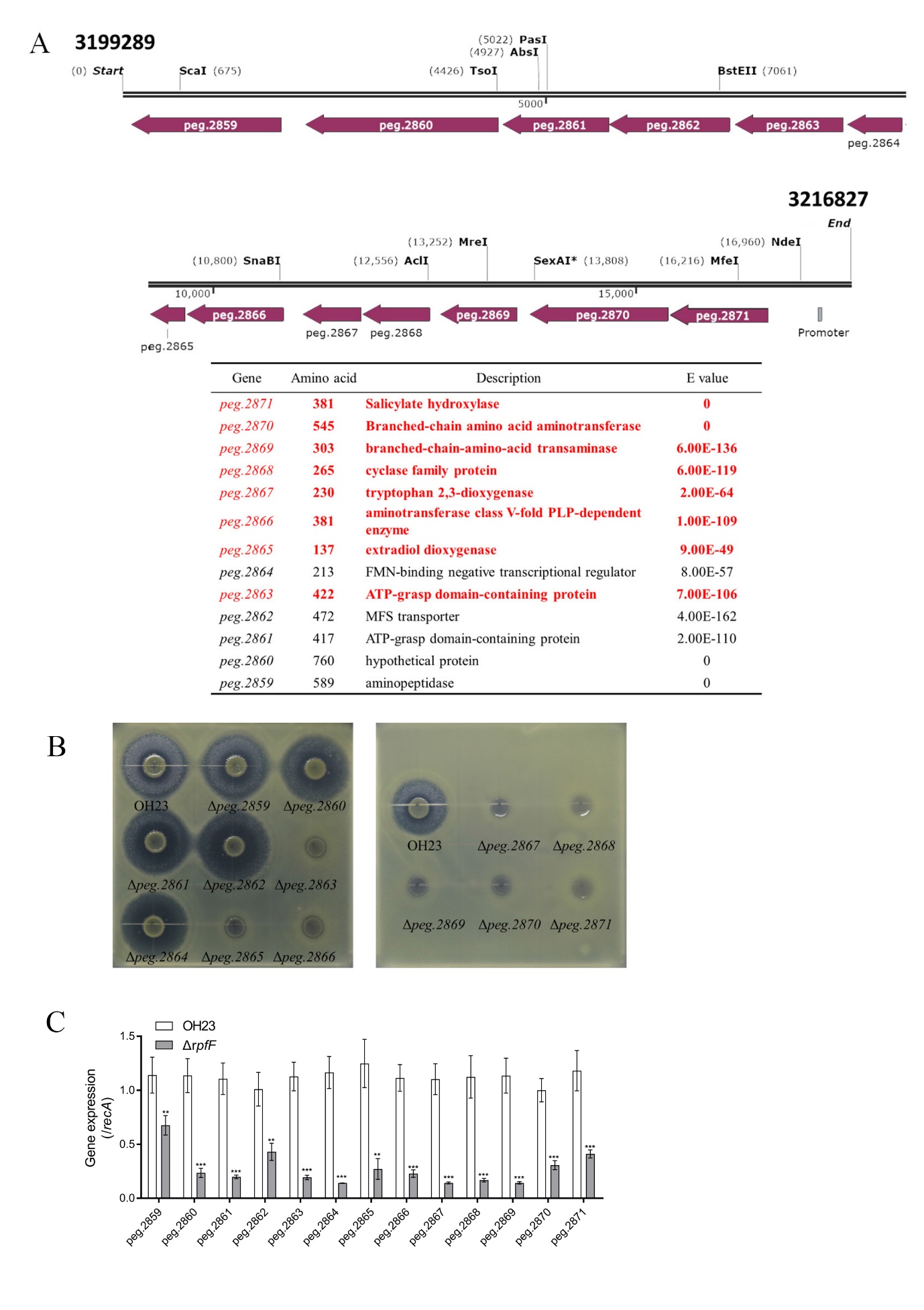


**Figure S8**. Preliminary map of the XSAC gene cluster and XSAC biosynthesis confirmation. (A) The map and function of ORFs related to the biosynthesis of XSAC. The genes highlighted in red were involved and indispensable in XSAC biosynthesis. (B) Determination of the activity of wild-type OH23 or mutants from the gene cluster to inhibit the growth of the plant pathogen *X. campestris* pv. *campestris* 8004. (C) The detection of gene expression from the XSAC gene cluster in wild-type OH23 and Δ*rpfF*. Different numbers of star (*) above the bars indicate a significant difference between the wild-type strain OH23 and mutant strains (ns: not sigificant; **: P < 0.01; ***: P < 0.001; ****: P < 0.000; t-test).

**
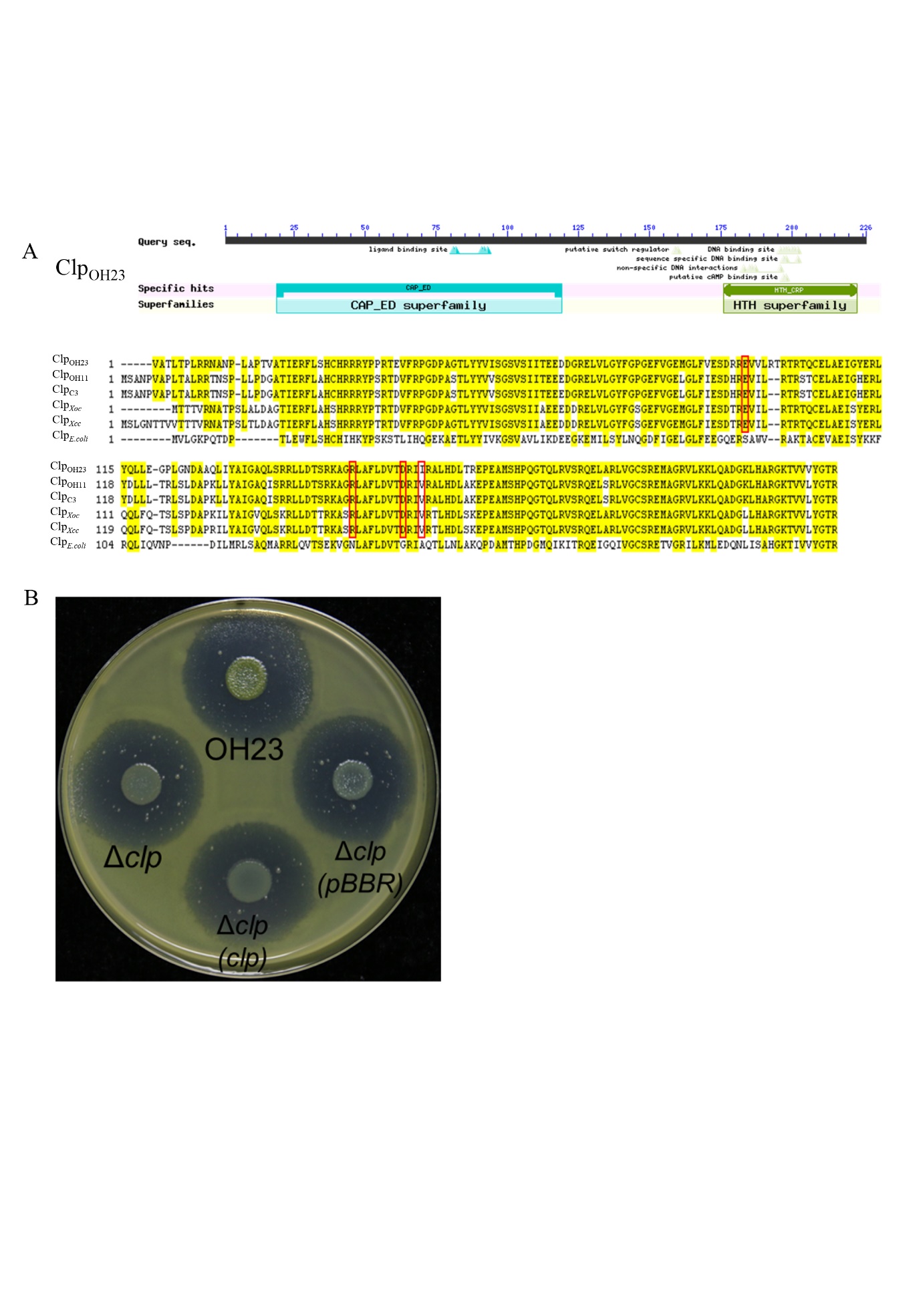
**

**Figure S9**. Identification of Clp in *L. brunescens*. (A) Domain perdition and comparison of Clp. Clp_OH11_, Clp_C3_, Clp*_Xoo_*, Clp*_Xcc_*, and Clp*_E.coli_* indicated the Clp proteins from *L. enzymogenes* OH11 ([Wang et al., 2014](#r34)), *L. enzymogenes* C3 (Kobayashi et al., 2005), *Xanthomonas oryzae* pv. *oryzae* PXO99A (NC_010717.2), *Xanthomonas campestris* pv. *campestris* str. ATCC 33913 (NC_003902.1), and *E. coli* str. K-12 substr. MG1655 (AAA58154.1). The red box indicates that these four amino acids were important for the binding of Clp to c-di-GMP in *Xanthomonas campestris* pv. *campestris* (Tao et al., 2010). (B) Determination of the activity of wild-type *L. brunescens* OH23, Δclp, and its complemented strains to inhibit the growth of the plant pathogen *X. oryzae* pv. *oryzae* RS105.

**Table S1**. Bacterial strains used in the Supplementary Material

| Strain/plasmid | Description | | Source or reference |
| --- | --- | --- | --- |
| *Lysobacter brunescens* | | |  |
| OH23 Rif | Spontaneous Rif^R^ mutant of OH23, Rif^R^ | |  |
| Δ*rpfF* | *rpfF* gene in-frame deletion mutant, Rif^R^ | | This study |
| Δ*peg.2859* | *peg.2859* gene in-frame deletion mutant | | This study |
| Δ*peg.2860* | *peg.2860* gene in-frame deletion mutant | | This study |
| Δ*peg.2861* | *peg.2861* gene in-frame deletion mutant | | This study |
| Δ*peg.2862* | *peg.2862* gene in-frame deletion mutant | | This study |
| Δ*peg.2863* | *peg.2863* gene in-frame deletion mutant | | This study |
| Δ*peg.2864* | *peg.2864* gene in-frame deletion mutant | | This study |
| Δ*peg.2865* | *peg.2865* gene in-frame deletion mutant | | This study |
| Δ*peg.2866* | *peg.2866* gene in-frame deletion mutant | | This study |
| Δ*peg.2867* | *peg.2867* gene in-frame deletion mutant | | This study |
| Δ*peg.2868* | *peg.2868* gene in-frame deletion mutant | | This study |
| Δ*peg.2869* | *peg.2869* gene in-frame deletion mutant | | This study |
| Δ*peg.2870* | *peg.2870* gene in-frame deletion mutant | | This study |
| Δ*peg.2871* | *peg.2871* gene in-frame deletion mutant | | This study |
| Δ*clp* | *clp* gene in-frame deletion mutant | | This study |
| Δ*clp* (*clp*) | Δ*clp* harboring pBBR-*clp* | | This study |
| Δclp (pBBR) | Δclp harboring pBBR1-MCS5 | | This study |
| *Xanthomonas* |  | |  |
| *Xanthomonas oryzae* pv. *oryzae* RS105 | Plant pathogen, causes bacterial leaf streak disease in rice | | ([Song et al., 2015](#r29)) |
| *Xanthomonas oryzae* pv. *oryzae* KACC10331 | | Plant pathogen, causes bacterial leaf blight disease in rice | ([Lee et al., 2005](#r15)) |
| *Xanthomonas campestris* pv. *campestris* 8004 | | Plant pathogen, causes bacterial black rot of crucifers | ([Qian et al., 2005](#r24)) |
| *Xanthomonas axonopodis* pv. *glycines* 12-2 | | Plant pathogen, causes bacterial soybean pustule | Lab strain |
| *Pseudomonas syringae* pv. *glycinea* PG4180 | | Plant pathogen, causes bacterial blight of soybean | Lab strain |
| *Acidovorax citrulli* DSM17060 | | Plant pathogen, causes seedling blight and bacterial fruit blotch of cucurbits | Lab strain |
| *Pseudomonas syringae* pv. *lachrymans* 814/98 | | Plant pathogen, causes angular leaf spot | Lab strain |
| *Erwinia amylovora* ATCC15580 | | Plant pathogen, causes fire blight in apple and pear trees | Lab strain |
| *E. coli* K-12 | | F´proA+B+ lacIq Δ(lacZ)M15 zzf::Tn10(TetR)/fhuA2 glnV Δ(lac-proAB) thi-1 Δ(hsdS-mcrB)5 | Lab strain |
| Pathogenic fungi | |  |  |
| *Botryosphaeria dothidea* | | Plant pathogen, causes botryosphaeria blight of pistachio and various woody plants | Lab strain |
| *Phytophthora capsica* | | Plant pathogen, causes blight and fruit rot of peppers and other important commercial crops | Lab strain |
| *Valsa ambiens* var. *Pyri* | | Plant pathogen, causes cytospora cankers of pear tree | Lab strain |
| *Colletotrichum gloeosporioide* | | Plant pathogen, causes rot of variety of crops | Lab strain |
| Plasmid |  | |  |
| pBBR1-MCS5 | Broad-host vector with a Plac promoter, Gm^R^ | | (Kovach et al., 1995) |
| pBBR-*clp* | pBBR1-MCS5 cloned with a 681 bp fragment of *clp* | | This study |
| pJQ-*peg.2859* | pJQ200SK derivative carrying two flanking fragments of *peg.2859*, Gm^R^ | | This study |
| pJQ-*peg.2860* | pJQ200SK derivative carrying two flanking fragments of *peg.2860*, Gm^R^ | | This study |
| pJQ-*peg.2861* | pJQ200SK derivative carrying two flanking fragments of *peg.2861*, Gm^R^ | | This study |
| pJQ-*peg.2862* | pJQ200SK derivative carrying two flanking fragments of *peg.2862*, Gm^R^ | | This study |
| pJQ-*peg.2863* | pJQ200SK derivative carrying two flanking fragments of *peg.2863*, Gm^R^ | | This study |
| pJQ-*peg.2864* | pJQ200SK derivative carrying two flanking fragments of *peg.2864*, Gm^R^ | | This study |
| pJQ-*peg.2865* | pJQ200SK derivative carrying two flanking fragments of *peg.2865*, Gm^R^ | | This study |
| pJQ-*peg.2866* | pJQ200SK derivative carrying two flanking fragments of *peg.2866*, Gm^R^ | | This study |
| pJQ-*peg.2867* | pJQ200SK derivative carrying two flanking fragments of *peg.2867*, Gm^R^ | | This study |
| pJQ-*peg.2868* | pJQ200SK derivative carrying two flanking fragments of *peg.2868*, Gm^R^ | | This study |
| pJQ-*peg.2869* | pJQ200SK derivative carrying two flanking fragments of *peg.2869*, Gm^R^ | | This study |
| pJQ-*peg.2870* | pJQ200SK derivative carrying two flanking fragments of *peg.2870*, Gm^R^ | | This study |
| pJQ-*peg.2871* | pJQ200SK derivative carrying two flanking fragments of *peg.2871*, Gm^R^ | | This study |
| pJQ-*clp* | pJQ200SK derivative carrying two flanking fragments of *clp*, Gm^R^ | | This study |

**Table S2**. Primers used in the Supplementary Material

| Primer name | Sequence (5′ → 3′) | Amplicon size (bp) | Usage |
| --- | --- | --- | --- |
| peg.2859-1 | CAGCCCGGGGGATCCGATGAGCGCAGGCGAACGCGAGCTGT | 523 | *peg.2859* detection |
| peg.2859-2 | GTACGATGTAAGCGCAGATCGACACGCTGAC |  |  |
| peg.2859-3 | CTGCGCTTACATCGTACGGTCTCGATATTTCCTG | 524 |  |
| peg.2859-4 | AGCGGCCGCTCTAGACAGCGCACCTTGTACGAACGGTT |  |  |
| peg.2860-1 | CAGCCCGGGGGATCCCGTTCCCGGCGCGCGGCCCTCGAACTCG | 523 | *peg.2860* detection |
| peg.2860-2 | GCCAAATGTGAGCGCCCCAGGCGATCGCCCCCGGTCG |  |  |
| peg.2860-3 | GCGCTCACATTTGGCTACGGTCCATGGTGGG | 527 |  |
| peg.2860-4 | GCGGCAGCGGCCGCTCTAGATCGAGGCGATCGAATCCGAAC |  |  |
| peg.2861-1 | CAGCCCGGGGGATCCTGGCCGGTCGGGTCCATGAAC | 523 | *peg.2861* detection |
| peg.2861-2 | CACGCATGTGATCCGGCGCCGAATCATC |  |  |
| peg.2861-3 | CGGATCACATGCGTGATTCAATGCCATG | 522 |  |
| peg.2861-4 | AGCGGCCGCTCTAGACGTCAACCTGGCCGGGGCGATCGTAC |  |  |
| peg.2862-1 | CAGCCCGGGGGATCCCCTCGCTGCCGGCGCCATGCGTCGGTTTC | 527 | *peg.2862* detection |
| peg.2862-2 | GCGATCGCGATGTGAATCACGCATGAAAATCCTGATCC |  |  |
| peg.2862-3 | GTGATTCACATCGCGATCGCATACGAGCAG | 528 |  |
| peg.2862-4 | GCGGCAGCGGCCGCTCTAGAGACGAGAATCGCGTCGTCCT |  |  |
| peg.2863-1 | GCAGCCCGGGGGATCCGGGAGCGCCACAGGAGGATCGC | 774 | *peg.2863* detection |
| peg.2863-2 | CCGCGATGTGACCCCCGCCGGCAACAG |  |  |
| peg.2863-3 | GGGGTCACATCGCGGGGAACTCCAGGTAT | 777 |  |
| peg.2863-4 | GCGGCAGCGGCCGCTCTAGAACAAGCTCAGGCGTGGCATGCAG |  |  |
| peg.2864-1 | GCAGCCCGGGGGATCCGCGTGGGTCTCCAGTTCTT | 524 | *peg.2864* detection |
| peg.2864-2 | CATCGATGTGAGGCGAACCAACCACCCTCCGCATG |  |  |
| peg.2864-3 | CGCCTCACATCGATGTTGGGCCTCGCGCGGCACTGC | 527 |  |
| peg.2864-4 | GCGGCAGCGGCCGCTCTAGACCCTGTCCACTGTCAACGACTGA |  |  |
| peg.2865-1 | TCCTGCAGCCCGGGGGATCCTGCGGCGCTTCATCGAC | 528 | *peg.2865* detection |
| peg.2865-2 | CTTCCATGACGCCCTGAACAAGCTCAGG |  |  |
| peg.2865-3 | AGGGCGTCATGGAAGGTTCCTTGGTTCAG | 523 |  |
| peg.2865-4 | CAGCGGCCGCTCTAGATGTCCAAGGCGCACGGCCTGGCCGGTG |  |  |
| peg.2866-1 | TCCTGCAGCCCGGGGGATCCTCGAAGAGCGGTTGTCTATGCA | 530 | *peg.2866* detection |
| peg.2866-2 | CGAAAACATGTGAACCAAGGAACCTTCCATGACCCTCGCAAGAG |  |  |
| peg.2866-3 | CTTGGTTCACATGTTTTCGGTTCCAGGTGTCGTGCGTCCTG | 529 |  |
| peg.2866-4 | GCGGCAGCGGCCGCTCTAGAAATGCGGGGCTTCCCGCAGCGAC |  |  |
| peg.2867-1 | TCCTGCAGCCCGGGGGATCCCCCAGCAGGACTTGGTCAGTCC | 528 | *peg.2867* detection |
| peg.2867-2 | GTCCTGTGTGAGCCTTGCTCCGCACATGCATGG |  |  |
| peg.2867-3 | AGGCTCACACAGGACATTCGTTCACCGCGTCGTCTC | 522 |  |
| peg.2867-4 | AGCGGCCGCTCTAGACAACGTCTCGTGGCGCCAGCGGTGGTC |  |  |
| peg.2868-1 | TCCTGCAGCCCGGGGGATCCCAGCGGCTGATCGTGACCACTTCGGAAG | 529 | *peg.2868* detection |
| peg.2868-2 | GCAACCATGTGAACGAATGTCCTGTGACGAACGAAGTGGCGAATC |  |  |
| peg.2868-3 | TTCGTTCACATGGTTGCGACAAGGTCGTGCGAGGCGCGTGCCT | 523 |  |
| peg.2868-4 | AGCGGCCGCTCTAGAGCGCGACGGCCGGCTCGTCACCC |  |  |
| peg.2869-1 | TCCTGCAGCCCGGGGGATCCAGGGATCAACAGCCTCTTCGGTG | 528 | *peg.2869* detection |
| peg.2869-2 | GCGTCGTGTGATGGGGAGCGCCCTGATCG |  |  |
| peg.2869-3 | CCCATCACACGACGCAGTCGTCCCATGGGACGAT | 523 |  |
| peg.2869-4 | CAGCGGCCGCTCTAGAATGCGTGTACTTCGCGAAGGATG |  |  |
| peg.2870-1 | TCCTGCAGCCCGGGGGATCCTCGAGCACGCCGCTCGTTCCT | 778 | *peg.2870* detection |
| peg.2870-2 | CCTGATGCCGGCGGGCCGCATGAGCGT |  |  |
| peg.2870-3 | CCCGCCGGCATCAGGAGACTTCCTCGATTTC | 777 |  |
| peg.2870-4 | GCGGCAGCGGCCGCTCTAGATTCAGCCAGGCGGATGACGACC |  |  |
| peg.2871-1 | TGCAGCCCGGGGGATCCCGAGCTGTTCACGCCATCGCACGCGATC | 527 | *peg.2871* detection |
| peg.2871-2 | TCACGAAATGTGATGCATACAGTCGCTCCTGTCGGGATC |  |  |
| peg.2871-3 | TATGCATCACATTTCGTGATAGGCTCGAATCACGGCATCG | 525 |  |
| peg.2871-4 | CAGCGGCCGCTCTAGAGTCGCCAACACGAGCATCCGAC |  |  |
| clp-1 | TGCAGCCCGGGGGATCCGGTCGTGCTCGGGTCAGTCGCCGAGCAG | 525 | *clp* detection |
| clp-2 | CCCCCGTGTGACCGGCCTCGCCTCCCGGTGA |  |  |
| clp-3 | CCGGTCACACGGGGGATGTGTTCATGCGAGCCAGAG | 523 |  |
| clp-4 | CAGCGGCCGCTCTAGAATCACCGGCTCCTCGGAGATGAG |  |  |
| pBBR-clp-1 | GCGGTGGCGGCCGCTCTAGATCAGCGGGTGCCGTAGACGAC | 724 | *clp* expression in pBBR1-MCS5 |
| pBBR-clp-2 | GGTCGACGGTATCGATAAGCTTCGTGGCCACGCTGACACCTCTC |  |  |
| RT- peg.2859-F | GATGCTCGTTGTAGTCCTTG | 165 | *peg.2859* expression detection |
| RT- peg.2859-R | CGCTGGTCGAGGAATCGAAC |  |  |
| RT- peg.2860-F | GCCAGCTCGCGCCCATGTAG | 187 | *peg.2860* expression detection |
| RT- peg.2860-R | TCGCAACTGCTTGCCATCTG |  |  |
| RT- peg.2861-F | CGAACAGTCCGGAGATGTAG | 234 | *peg.2861* expression detection |
| RT- peg.2861-R | AGTATTGCGGCTGGTATGTC |  |  |
| RT- peg.2862-F | GATGCCGAGTGCGGATGTTG | 176 | *peg.2862* expression detection |
| RT- peg.2862-R | GATCGCCCGTGAACTGATGG |  |  |
| RT- peg.2863-F | TGCTCGCTGAGGAACCCATC | 133 | *peg.2863* expression detection |
| RT- peg.2863-R | ATTGCGTGCAGACGATCTAC |  |  |
| RT- peg.2864-F | CGACGATGCCTTGTCGATCC | 183 | *peg.2864* expression detection |
| RT- peg.2864-R | GCAGCATGACCGATTACTTC |  |  |
| RT- peg.2865-F | AAACGGATGTTCCCGTAACC | 151 | *peg.2865* expression detection |
| RT- peg.2865-R | ATCGCCACTTCGGGCTTGTC |  |  |
| RT-peg.2866-F | CGGCCGTCTTCACTTTCTGG | 159 | *peg.2866* expression detection |
| RT- peg.2866-R | TCACGCCGGATGAGATGGTC |  |  |
| RT- peg.2867-F | AGTGCCGATGGTGCGTACTG | 130 | *peg.2867* expression detection |
| RT- peg.2867-R | GCAGGAAATCTTCCGAAGTG |  |  |
| RT- peg.2868-F | CGGGAGCAATTCGAGATTTC | 158 | *peg.2868* expression detection |
| RT- peg.2868-R | TGGCTGAGGACACCTCAATC |  |  |
| RT- peg.2869-F | CACCGTGGCATCGAAGACTG | 136 | *peg.2869* expression detection |
| RT- peg.2869-R | TCTCGCAGACGAGTTGTTTC |  |  |
| RT- peg.2870-F | GGTCGTTGCTGGTGAGGATG | 106 | *peg.2870* expression detection |
| RT- peg.2870-R | GAACAGACTGCGAACGTACC |  |  |
| RT- peg.2871-F | AAGACCGGGATGGTTCTGAC | 94 | *peg.2871* expression detection |
| RT- peg.2871-R | ACGGCATCGCGATCAAGATG |  |  |
| RT-clp-F | GAGCGTTTCCTGTCCCACTG | 101 | *clp* expression detection |
| RT-clp-R | CCGGAGATCACGTAATACAA |  |  |
| RT-recA-F | GTCACCGAAATCCTCTATGG | 164 | *recA* expression detection |
| RT-recA-R | GGGTTGTCCTTCATGTACTG |  |  |

**References used in Supplementary Material**

Kobayashi D. Y., Reedy R. M., Palumbo J. D., et al (2005). A clp gene homologue belonging to the Crp gene family globally regulates lytic enzyme production, antimicrobial activity, and biological control activity expressed by *Lysobacter enzymogenes* strain C3. *Appl Environ Microb*. 71(1):261-269.

Lee, B. M., Park, Y. J., Park, D. S., Kang, H. W., Kim, J. G., Song, E. S., et al. (2005). The genome sequence of *Xanthomonas oryzae* pathovar *oryzae* KACC10331, the bacterial blight pathogen of rice. *Nucleic Acids Res*. 33(2):577-586.

Qian, W., Jia, Y., Ren, S. X., He, Y. Q., Feng, J. X., Lu, L. F., et al. (2005). Comparative and functional genomic analyses of the pathogenicity of phytopathogen *Xanthomonas campestris* pv. *campestris*. *Genome Res*. 15(6):757-767.

Song, Z., Zhao, Y. C., Zhou, X. Y., Wu, G., Zhang, Y., Qian, G., et al. (2015). Identification and characterization of two novel DSF-controlled virulence associated genes within the *nodB*-*rhgB* locus of *Xanthomonas oryzae* pv. *oryzicola* Rs105. *Phytopathology*. 105(5):588-596.

Tao F., He Y. W., Wu D. H., et al. (2010). The cyclic nucleotide monophosphate domain of *Xanthomonas campestris* global regulator Clp defines a new class of cyclic di-GMP effectors. *J Bacteriol*. 192(4):1020-1029.

Wang Y., Zhao Y., Zhang J., et al. (2014). Transcriptomic analysis reveals new regulatory roles of Clp signaling in secondary metabolite biosynthesis and surface motility in *Lysobacter enzymogenes* OH11. *Appl Microbiol Biot*. 98(21):9009-9020.
